# Statistical modeling and significance estimation of multi-way chromatin contacts with HyperloopFinder

**DOI:** 10.1093/bib/bbae341

**Published:** 2024-07-14

**Authors:** Weibing Wang, Yusen Ye, Lin Gao

**Affiliations:** Department of Computer Science, School of Computer Science and Technology, Xidian University, Xi’an, Shaanxi, China; Department of Computer Science, School of Computer Science and Technology, Xidian University, Xi’an, Shaanxi, China; Department of Computer Science, School of Computer Science and Technology, Xidian University, Xi’an, Shaanxi, China

**Keywords:** 3D genome, chromatin loops, multi-way chromatin contacts

## Abstract

Recent advances in chromatin conformation capture technologies, such as SPRITE and Pore-C, have enabled the detection of simultaneous contacts among multiple chromatin loci. This has made it possible to investigate the cooperative transcriptional regulation involving multiple genes and regulatory elements at the resolution of a single molecule. However, these technologies are unavoidably subject to the random polymer looping effect and technical biases, making it challenging to distinguish genuine regulatory relationships directly from random polymer interactions. Here, we present HyperloopFinder, a method for identifying regulatory multi-way chromatin contacts (hyperloops) by jointly modeling the random polymer looping effect and technical biases to estimate the statistical significance of multi-way contacts. The results show that our model can accurately estimate the expected interaction frequency of multi-way contacts based on the distance distribution of pairwise contacts, revealing that most multi-way contacts can be formed by randomly linking the pairwise contacts adjacent to each other. Moreover, we observed the spatial colocalization of the interaction sites of hyperloops from image-based data. Our results also revealed that hyperloops can function as scaffolds for the cooperation among multiple genes and regulatory elements. In summary, our work contributes novel insights into higher-order chromatin structures and functions and has the potential to enhance our understanding of transcriptional regulation and other cellular processes.

## Introduction

Genomes encode genetic information on linear DNA sequences, but proper expression of genes require chromatin to adopt complex three-dimensional (3D) spatial structures at different genomic scales, including chromosome territories [1], A/B compartments [1], topologically associating domains (TADs) [2], and chromatin loops [3–5]. Chromatin loops play a critical role in gene regulation and have been extensively studied, encompassing CTCF loops, enhancer-promoter loops, gene loops, and Polycomb-mediated loops [6]. Chromatin conformation capture techniques such as Hi-C [1, 2, 4] and ChIA-PET [3, 5, 7, 8], resolve the higher-order structure of the 3D genome by capturing the contact frequency between any two chromatin loci, facilitating the genome-wide detection of chromatin loops. Several computational methods have been proposed for loop detection in Hi-C data, such as HiCCUPS [4] and Fit-Hi-C [9, 10], as well as for loop detection in ChIA-PET data, such as ChIA-PET2 [11] and ChIA-PET Tool [12].

However, we could not directly obtain simultaneous contacts of more than two loci from the Hi-C or ChIA-PET data [13–18]. Specifically, three chromatin loci A, B, and C can form three loops: AB, BC, and AC, but we cannot simply conclude that these three loci physically interact with each other in the bulk-level data. Because A and B may form loops in some cells, and B and C or A and C may form loops in other cells. Merging all the pairwise contacts from different cells may lead to an incorrect conclusion that the three sites are spatially colocalized. Despite the population-average problem, several studies have found multi-way chromatin interaction patterns from Hi-C or ChIA-PET data. Rao et al. observed the transitivity of adjacent loops, meaning that if loop anchors A and B form a loop, B and C form a loop, then A and C also form a loop, suggesting spatial colocalization of these three loop anchors [4]. Sanborn et al. observed that multiple chromatin loops can form an isolated clique in a loop network [19]. Li et al. proposed a multigene interaction model, suggesting multiple genes are spatially colocalized and form a transcription factory to transcript collaboratively [3]. These works highlight the urgent need for sequencing technologies capable of simultaneously capturing interactions among multiple loci.

Recent advances in chromatin conformation techniques, such as SPRITE [13] and Pore-C [17], have enabled the detection of simultaneous interactions among multiple chromatin loci at the whole-genome level. SPRITE utilizes unique tag sequences to label chromatin fragments associated with the same complex. This allows for the distinction of different multi-way chromatin contacts by high-throughput sequencing. Through SPRITE, researchers have unveiled various multi-way interaction patterns within the nucleus, including interactions within A/B compartments, interactions among gene clusters, and interactions involving consecutive CTCF loops. Later, based on SPRITE, researchers developed a single-cell multi-way chromatin interaction capture technology [20] and a specific protein-enriched multi-way chromatin interaction capture technology [21]. Additionally, Pore-C [17] and HiPore-C [22] are developed to capture multi-way contacts by nanopore long-read sequencing of proximally ligated chromatin concatemers, while ChIA-Drop captures multi-way chromatin interactions via droplet-based and barcode-linked sequencing [14].

The development of these technologies presents new opportunities and challenges in comprehending the higher-order chromatin structure within the nucleus. Meanwhile, some novel tools for processing and analyzing multi-way chromatin interaction data have emerged. MIA-Sig [23] is a novel algorithm framework that utilizes signal processing and information theory to denoise ChIA-Drop [14] data. MATCHA [24] is a computational framework that leverages hypergraph representation learning to enhance the data quality of SPRITE and ChIA-Drop data [14]. Furthermore, hypergraph theory is employed for the data representation and analysis of multi-way contacts [25].

However, computational methods for detecting regulatory multi-way chromatin contacts, called hyperloops, remain significantly underexplored. Specifically, these sequencing technologies are unavoidably subject to the random polymer looping effect and technical biases, yielding random multi-way chromatin contacts, rendering it challenging to distinguish genuine regulatory relationships from random polymer interactions. The term ‘hyperloop’ is derived from the mathematical concept of hyperedge in graph theory. A hyperedge is a generalization of an edge that connects more than two nodes simultaneously [26]. Similarly, a hyperloop is a generalization of a loop that connects more than two genomic loci simultaneously.

In this study, we defined hyperloops and proposed an effective and efficient method, HyperloopFinder, to detect hyperloops by modeling it as a frequent pattern mining problem and establishing a binomial model to estimate the statistical significance. To efficiently detect frequent multi-way contacts as candidates of hyperloops, we employed the FP-growth algorithm [27, 28]. Low-frequency interactions are usually noise from random connections and have no biological significance. Therefore, filtering them out at the initial stage can significantly reduce the number of candidates. The binomial model jointly models the random polymer looping effect of multiple chromatin loci and technical biases of SPRITE and Pore-C data to predict the expected frequency of randomly ligated multi-way contacts and estimate their statistical significance. To reduce the search space, boost the search speed, and enhance interpretability, we detected hyperloops that are connected by pairwise chromatin loops.

The results demonstrate that our model can accurately estimate the expected frequency of multi-way contacts based on the distance distribution of pairwise contacts, revealing that most multi-way contacts are formed by randomly linking adjacent pairwise contacts and that linear genomic distance is a major determinant of interaction frequency of multi-way contacts. This further suggests that only using interaction frequency to measure the importance of multi-way contacts is not enough and will bring many false positives. Subsequently, we examined the influence of technical biases, such as GC content and mappability [29–31], on the interaction frequency of multi-way contacts and found that our model achieves greater accuracy in predicting interaction frequency when considering these technical biases. Next, we validated the spatial colocalization of the interaction sites of hyperloops using image-based DNA seqFISH+ data [32]. Our results indicate that the hyperloops detected by our method exhibit a higher colocalization ratio than randomly generated control samples. Subsequently, we analyzed the relationship between the hyperloops and other higher-order chromatin structures like TADs and chromatin A/B compartments. Our analysis revealed that the majority of hyperloops are within a single TAD, and their anchors are typically located in the open chromatin regions. Lastly, we investigated the relationship between hyperloops and gene regulation, uncovering a significant association between hyperloops and multigene chromatin complexes. Moreover, genes belonging to multigene hyperloops are usually characterized by high expression and broad expression in multiple tissues. In summary, our study offers novel insights into higher-order chromatin structures and functions, potentially enhancing our comprehension of transcriptional regulation and other cellular processes.

## Results

### Overview of HyperloopFinder

We defined hyperloops as multi-way chromatin contacts that are statistically significant and connected by pairwise chromatin loops. The term ‘significant’ means that the contact frequency of each hyperloop is significantly higher than its background contact frequency driven by the random polymer looping effect and technical biases. Additionally, the term ‘connected by pairwise chromatin loops’ indicates that hyperloops are higher-order structures incorporating multiple specific pairwise loops, such as enhancer-promoter loops and CTCF loops.

**Figure 1:**
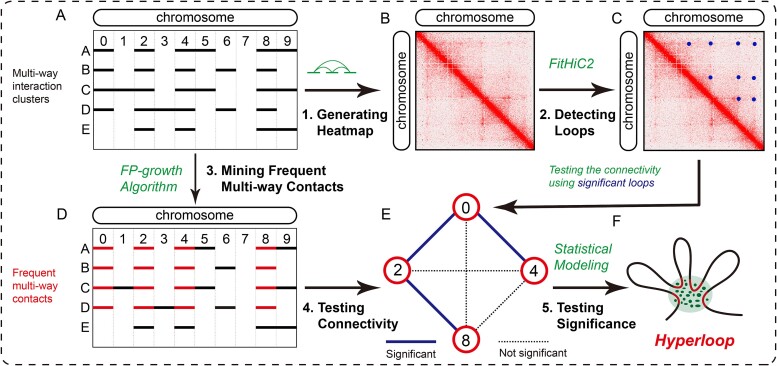
Overview of HyperloopFinder. (A) Examples of multi-way interaction clusters. Bold solid lines represent the interacting sites of clusters A–E. (B) Hi-C-like heatmap generated from multi-way interaction data. (C) Schematic illustration of pairwise loops that were detected with FitHiC2. (D) An example of frequent multiway interaction {0, 2, 4, 8} that is supported by four clusters: A–D. (E) Graph form of hyperloop candidate. Nodes represent bins of hyperloop, and edges represent the pairwise decomposition of the hyperloop. Bold solid lines represent the significant loops detected by step 2. (F) Schematic illustration of hyperloops.

To detect hyperloops efficiently and effectively, we proposed HyperloopFinder. The main steps are as follows (Fig. 1): Step 1: Generating a Hi-C-like heatmap from multi-way interaction clusters. SPRITE or Pore-C captures multi-way chromatin interactions, whereas Hi-C captures pairwise contacts. To detect loops from multi-way interaction clusters using tools developed for Hi-C data, we first decomposed multi-way contacts into pairwise contacts. Specifically, we enumerated all pairwise contacts within a SPRITE or Pore-C cluster. Subsequently, genomic regions of each chromosome were split into bins of equal length, just like Hi-C, and each interaction site within multi-way clusters was assigned to corresponding bins. In this way, a contact matrix is constructed for each chromosome. Step 2: Detecting loops from the Hi-C-like interaction matrix. Next, we can detect loops from the pairwise interaction matrix using FitHiC2 [9, 10], which is designed to detect statistically significant chromatin contacts from Hi-C data by fitting the relationship between genomic distance and contact probability and modeling the contact frequency through a binomial model. Step 3: Mining frequent multi-way contacts using the FP-growth algorithm. Subsequently, we determined the frequency of multi-way contacts using the FP-growth algorithm known for its efficiency in mining frequent item sets [27, 28]. Consequently, this algorithm enables efficient enumeration of all frequent multi-way contacts with frequencies surpassing the minimum support threshold. Step 4: Testing connectivity. Then, we tested whether the multi-way contacts are connected by the loops detected in Step 2. The following observations justify the need for this limitation. If multiple chromatin fragments are colocalized to form a hyperloop, a pairwise loop exists between any two fragments. Therefore, the presence of loops between any pair of interaction loci is a necessary but not sufficient condition for the existence of a hyperloop. However, we relaxed this stringent constraint and only required that the loci of the hyperloops be connected. This constraint offers two advantages: Firstly, we can focus on hyperloops that are only constructed by functional chromatin loops such as enhancer-promoter loops and CTCF loops. Secondly, this step can reduce the number of hyperloop candidates and reduce the time consumption in the next step. Step 5: Testing significance. Due to the influence of the random polymer looping effect and technical biases, it is challenging to distinguish regulatory multi-way contacts from random polymer contacts. Therefore, it is necessary to estimate the expected interaction frequencies of the multi-way contacts and then, as a control, assess the significance of each hyperloop candidate. Details of these steps are described in the Methods section.

### Statistical modeling of multi-way chromatin contacts

**Figure 2:**
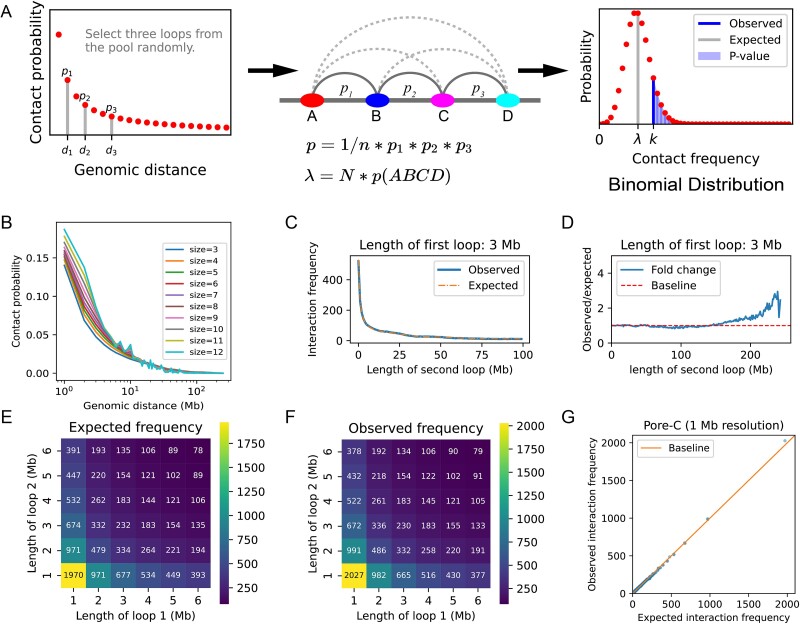
Statistical modeling and significance estimation of multi-way chromatin contacts. (A) Schematic of the background model. (B) Genomic distance distribution of pairwise contacts for different sizes of multi-way contacts. (C) Fixed the length of the first loop to 3 MB, observed and expected interaction frequency all decreased with the increase of the length of the second loop. (D) Fixed the length of the first loop to 3 MB, fold enrichment between observed and expected interaction frequency with the increase of the length of the second loop. (E) Heatmap of expected interaction frequency for different length pairs of loops. (F) Heatmap of observed interaction frequency for different length pairs of loops. (G) Expected interaction frequencies are highly correlated with observed interaction frequencies.

We estimated the expected interaction frequency of hyperloops from the expected frequency of pairwise interactions using a probabilistic model. Assuming that random multi-way contacts are formed by random combinations of pairwise contacts of different lengths, we proposed a model to simulate random multi-way contacts (Fig. 2A and Supplementary Figure S1 available online at http://bib.oxfordjournals.org/): Firstly, molecular complexes that link multiple chromatin fragments were decomposed into pairwise contacts, and then we obtained the probability distribution of the genomic distance of pairwise contacts (left of Fig. 2A and Fig. 2B). To generate a multi-way contact involving four chromatin loci, we randomly sampled a start point and three loop length, and then connected them sequentially (illustrated in the middle of Fig. 2A). Given the assumption that the start point and loop length are independent random variables, the probability of generating the hyperloop can be computed as the product of the sampling probability of the start point and the sampling probability of the length of its composed loops (middle of Fig. 2A). Subsequently, we calculated the expected interaction frequency through multiplying the probability by the number of experiments. The number of experiments is a key parameter that needs to be learned from the raw multi-way interaction data. See the methods section for more details. Here, we did not use the usual regression model to predict the expected interaction frequency, because we believe that a simple model like ours is predictive enough and simple enough and can reveal the relationship between pairwise contacts and multi-way contacts. Finally, statistical significance estimation employs a binomial model (right of Fig. 2A, more details in Methods section).

### Linear genomic distance is a major determinant of the interaction frequency of multi-way contacts

To evaluate the capability of our model for estimating the expected interaction frequency of Pore-C data at a specific combination of loop lengths, we counted the frequency of all 3-way contacts at 1 MB resolution, deriving the mean frequency for each combination of loop lengths. We used 1 MB resolution because it is difficult to enumerate all multi-way interaction candidates when the resolution is too high. Concurrently, we computed the expected interaction frequency of all 3-way contacts using our background model. For 3-way contacts, when we held the length of the first loop constant at 3 MB, both observed and expected interaction frequencies declined as the length of the second loop increased (Fig. 2C), and the observed and expected values align notably well within the range of 150 MB length (Fig. 2D). When the lengths of the two constituent loops differ significantly, the interaction frequencies of hyperloops tend to increase. This trend is apparent when the total length of the two loops for each 3-way contact remains fixed, as discernible from both the heatmaps of expected and observed interaction frequencies (Fig. 2E–F, Supplementary Figure S2 available online at http://bib.oxfordjournals.org/). Lastly, we observed that expected and observed interaction frequencies are highly correlated and unbiased (Fig. 2G). In addition, our model exhibits a strong fit with the SPRITE data (Supplementary Figures S3–S6 available online at http://bib.oxfordjournals.org/).

Overall, our background model can estimate the expected interaction frequency of multi-way contacts and reveals that the linear genomic distance is a major determinant of the frequency of multi-way contacts. This observation suggests that random combinations of pairwise contacts alone may give rise to multi-way contacts with notably elevated frequencies. Consequently, it becomes increasingly evident that relying solely on interaction frequency to evaluate the importance of hyperloops is insufficient, as this approach will yield numerous false positives.

### Modeling with technical biases can improve the prediction of the interaction frequency

**Figure 3:**
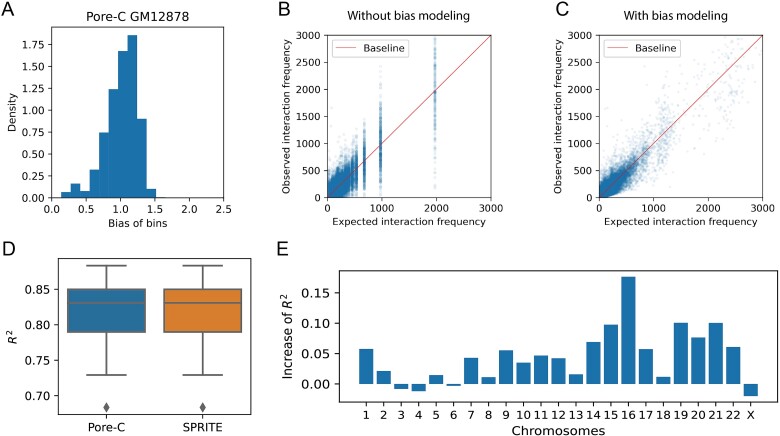
Modeling with the biases of Pore-C data. (A) Histogram showing the distribution of biases of bins, GM12878 cell line, Pore-C data, chr1. (B) Expected interaction frequency vs observed interaction frequency without bias modeling. (C) Expected interaction frequency vs observed interaction frequency with bias modeling. (D) Boxplots showing the *R*^2^ distribution of 23 chromosomes. *R*^2^ is used to evaluate the goodness-of-fit between the observed and predicted values in the regression task. In this place, we regard the expected frequency as predicted values. (E) Bar plot showing the increase in *R*^2^ after modeling the bias.

The frequency of multi-way contacts is influenced by some technical biases, such as GC content and mappability, just like Hi-C data. To estimate the expected interaction frequency more accurately, we applied matrix balance theory to estimate the bias value of each bin of Pore-C data (Fig. 3A) and then multiplied the calculated expected interaction frequency by the bias values of all interaction bins as the final expected frequency of the multi-way contacts.

Results indicate that the expected interaction frequency after correction becomes more continuous and aligns more closely with the observed interaction frequency (Fig. 3B–C). Viewing the calculation of the expected interaction frequency as a regression problem enables us to evaluate the accuracy of the expected value by evaluating the goodness-of-fit of the regression. The coefficient of determination *R*^2^ is a typical statistical measure to indicate the proportion of variance that the model explains in the observed data. Results reveal that the median *R*^2^ value of the expected interaction frequency versus the observed interaction frequency is ~0.83 for both Pore-C data and SPRITE data in the GM12878 cell line at 1 MB resolution, suggesting that the random polymer looping effect and the technical biases on the bins can explain 83% of the variance in the observed data (Fig. 3D). We also compared the *R*^2^ before and after correction, revealing an increase in the values for most chromosomes within Pore-C data (Fig. 3E), as well as all chromosomes within SPRITE data (Supplementary Figures S7–S8 available online at http://bib.oxfordjournals.org/). Notably, in the case of chr16 within SPRITE data, which exhibited the most significant increase, the value rose by over 20% (Supplementary Figure S7D available online at http://bib.oxfordjournals.org/). To prevent the calculation of *R*^2^ from being affected by outliers and heterogeneity of residual variances, we log-transformed the expected and observed values and then calculated the *R*^2^. The results show that bias modeling can significantly improve the accuracy of prediction for both SPRITE data and Pore-C data (p-value <9.6e-07, by Wilcoxon Rank-Sum Test, Supplementary Figure S9 available online at http://bib.oxfordjournals.org/).

Overall, HyperloopFinder models technical biases for Pore-C and SPRITE data to provide accurate predictions of the expected frequency of multi-way contacts. The results further suggest that the random polymer looping effect and technical bias determine most of the variance in the frequency of multi-way contacts. In addition, our method can effectively mitigate technical biases in multi-way interaction data before data analysis.

### Evaluation of HyperloopFinder

Resolution is one of the most important parameters for HyperloopFinder. In our experiments, we used resolutions of 10 kb and 25 kb, based on the following considerations: The 10 kb resolution enables us to uncover more detailed multi-way regulatory relationships, while the 25 kb resolution provides a more comprehensive result. Prior work [33] suggests that the median length of the human gene is around 24 kb, so resolutions exceeding 25 kb might hinder our ability to identify gene-level regulatory relationships. To illustrate the impact of resolution settings on our methods, we evaluated our method at 10 kb, 20 kb, 50 kb, and 100 kb resolution (Supplementary Tables 1–3). The results indicate that at 10 kb resolution, only 11 851 hyperloops were detected in the GM12878 Pore-C data, with the majority being 3-hyperloops (97%) and only 359 being 4-hyperloops. However, at a 25 kb resolution, 350 303 hyperloops were detected. Further reduction in resolution would detect hyperloops involving more sites.

We also compared the results from different datasets using the overlap coefficient which is defined as the size of the intersection divided by the smaller of the two set sizes. We used the overlap coefficient because the results obtained from low-resolution data typically yield more results than those from high-resolution data. The results show that the hyperloops found at different resolutions have a strong overlap, with an average overlap coefficient of 0.96 and a minimum overlap coefficient of 0.82 (Supplementary Table 4).

**Figure 4:**
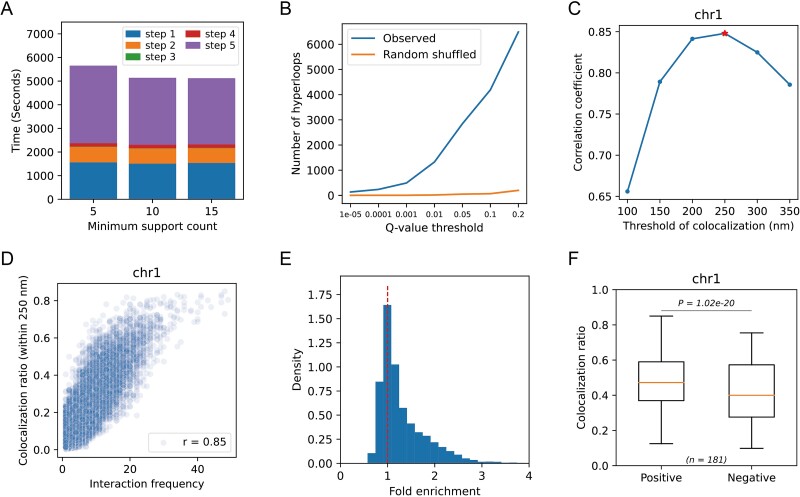
Evaluation of HyperloopFinder. (A) Running time of HyperloopFinder for different steps and minimum support count setting. GM12878 cell line, Pore-C data, chr1, and 10 kb resolution are used. step 1: Generating hi-C heatmap, computing the biases of bins, and detecting pairwise loops. Step 2: Binning and splitting the multi-way contacts to different chromosome files. Step 3: Mining frequency pattern using FP-growth algorithm. step 4: Testing the connectivity of hyperloop candidates using pairwise loops detecting from step 1. Step 5: Testing the significance of hyperloop candidates. (B) The numbers of hyperloops are shown in different q-value thresholds, compared with the random shuffled Pore-C data. This plot shows the low false discovery rate of HyperloopFinder. 25 kb resolution, GM12878 cell line, chr21. (C) The correlation coefficient between the interaction frequency of SPRITE data and the colocalization ratio of DNA seqFish+ data at different colocalization thresholds. The star represents the highest points. 25 kb resolution, mESC cell line. (D) The scatter plot shows the relationship between the interaction frequency of SPRITE data and the colocalization ratio of DNA seqFish+ data with the threshold of colocalization setting to 250 nm. (E) Fold enrichment of colocalization ratio of hyperloop interacting sites compared with random shuffled samples. (F) Boxplot shows the distribution difference of colocalization ratio between hyperloops and control samples where n is the number of hyperloops.

HyperloopFinder employs GNU Parallel software [34] for the parallel detection of hyperloops across various chromosomes, significantly enhancing algorithmic efficiency. We evaluated the running efficiency of HyperloopFinder, and the results show that it could be completed within 10 min on Pore-C data with a 10 kb resolution in the GM12878 cell line. Notably, most of the time was dedicated to constructing and evaluating the background model (Fig. 4A, Supplementary Figure S10 available online at http://bib.oxfordjournals.org/). Although mining frequent patterns takes only a small amount of time, the time taken for estimating the significance of patterns will directly depend on the number of candidate frequent patterns. Therefore, it is necessary to evaluate only frequent and connected patterns rather than all.

Then, we evaluated the false discovery rate of HyperloopFinder. A shuffled dataset was generated by randomly shuffling the start points of multi-way chromatin clusters while maintaining a consistent distance distribution. The results show that only a small number of hyperloops were detected in a randomly shuffled dataset compared to the observed dataset (Fig. 4B). This indicates that HyperloopFinder detects hyperloops with an extremely low false discovery rate (Supplementary Figure S11 available online at http://bib.oxfordjournals.org/).

Then, we used DNA seqFISH+ data of the mESC cell line [32] to evaluate hyperloops detected by HyperloopFinder. DNA seqFISH+ enables the imaging of thousands of targeted DNA loci in single cells and achieves a resolution of up to 25 kb in a local region. We used a greedy strategy to divide each fluorescence site into different chromosomes, and ~50% of the missing loci were completed using linear interpolation, resulting in the spatial position coordinates of the consecutive DNA loci for each chromosome within 1.5 MB-sized region at 25 kb resolution (Supplementary Text).

For each *3-hyperloop* at 25 kb resolution, we computed the average distance among three interaction loci. If the average distance of three sites is less than a distance threshold $maxdist$, we regarded these three sites as spatially colocalized. To determine the value of $maxdist$, we computed the correlation coefficient of the frequency of all multi-way contacts and the ratio of the three loci co-located in all cells at different $maxdist$. The correlation coefficient reached its maximum value (0.85) for chr1 at a distance threshold of 250 nm (Fig. 4C, Supplementary Figure S12 available online at http://bib.oxfordjournals.org/). Therefore, in subsequent analyses, we considered DNA loci with an average distance of less than 250 nm as colocalization. The higher the interaction frequency of the hyperloops, the higher the colocation ratio of the interaction sites (Fig. 4D), which reveals that it is reasonable to measure the importance of hyperloops using the interaction frequency. However, a high interaction frequency could merely indicate close linear genomic proximity of multiple DNA loci, influenced by distance effects. To eliminate the bias caused by the distance effect, we generated negative samples with the same distance distribution for each hyperloop and compared the colocalization ratio of their corresponding fluorescence sites. The results show that the hyperloops detected by our method have a higher colocalization ratio than randomly generated negative ones for most chromosomes (Fig. 4E–F, Supplementary Figure S13 available online at http://bib.oxfordjournals.org/).

### Hyperloops and transcriptional regulation

**Figure 5:**
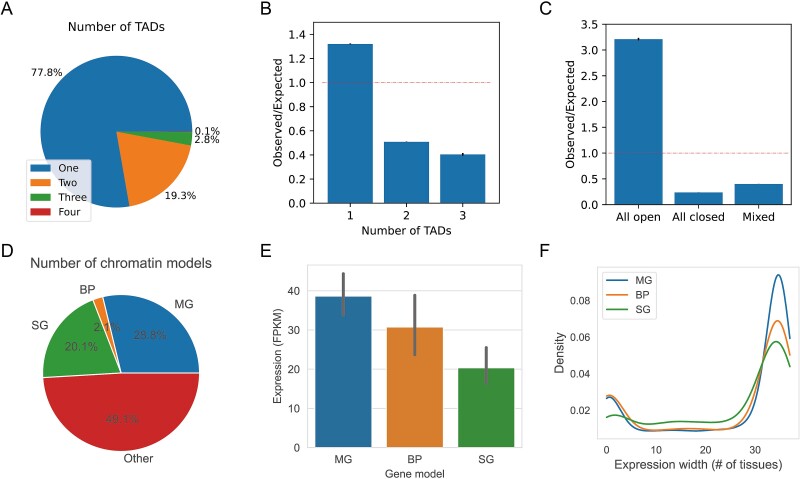
Hyperloops and transcriptional regulation. (A) Pie chart shows the percentage of hyperloops that span different TAD numbers. (B) Fold enrichment of the number of hyperloops that span different TAD numbers compared with random shuffled TADs. (C) Fold enrichment of the number of hyperloops that span different compartment patterns compared with randomly shuffled compartment labels. (D) Pie chart shows the percentage of hyperloops that belong to different chromatin models. MG represents the multiple gene hyperloops, BP represents the basal promoter hyperloops, SG represents the SG, and other represents hyperloops that are not associated with gene expression. (E) Bar plots show the expression difference of genes associated with different chromatin models. (F) Expression width analysis of the genes that belong to different chromatin models.

Next, we examined the relationship between hyperloops and other higher-order chromatin structures like TADs and compartments. For comparison with randomized scenarios, the starting loci of the TADs from the GM12878 cell line were randomly shuffled, and 20 random groups of TADs were generated as control groups. The results show that the majority of hyperloops at 25 kb resolution detected from SPRITE data (77.8%) are situated within a single TAD (1.3x enrichment). This concurs with the notion that TADs commonly serve as regulatory units (Fig. 5A–B). Moreover, anchors of hyperloops at 25 kb resolution are usually located in the open chromatin region (3.2x enrichment). This is consistent with the observation that chromatin openness is a prerequisite for transcriptional regulation and gene expression (Fig. 5C). The results are consistent when using mESC SPRITE data (Supplementary Figure S14 available online at http://bib.oxfordjournals.org/). These results are consistent with recent studies on multi-way chromatin contacts [16].

Then, we explored the relationship between hyperloops and gene regulation. We annotated anchors of hyperloops from mESC SPRITE data at 25 kb resolution as enhancers or promoters based on their overlap with corresponding regulatory elements marked by chromHMM [35]. Based on the number of expressed genes and enhancers involved in hyperloop anchors, we divided hyperloops into four types: single gene hyperloops (SG), multigene hyperloops (MG), basal promoter hyperloops (BP), and others. Single gene hyperloops involve the interaction of one gene and multiple enhancers, while multigene hyperloops involve the interaction of multiple genes. Basal hyperloops involve one gene but no enhancer, while others mean no gene involved. This classification was inspired by Li’s work [3], which observed these interaction patterns from ChIA-PET data of population cells. In our work, we tried to use multi-way interaction data to re-examine this classification. The results show that most hyperloops are other types (49.1%), multigene hyperloops account for 28.8%, SG account for 20.1%, and basal promoter hyperloops only account for 2.1% (Fig. 5D). We divided all expressed genes into three types based on the type of hyperloop involved: BP gene, SG gene, and MG gene. The results show that 68.2% of genes are involved in MG hyperloops, greatly surpassing the involvement in other gene types, which is consistent with the results observed in previous work (Supplementary Figure S15 available online at http://bib.oxfordjournals.org/) [3]. We then compared the differences in the expression of these three gene types. The results show that the MG gene usually has a higher expression than the SG gene (Fig. 5E).

We then analyzed gene expression width on these three gene types using 38 distinct cell types. Genes expressed across most cell types are typically categorized as housekeeping genes, whereas genes expressed in only a limited number of cell types are designated as cell type-specific genes. The results show that the expression width of the gene shows two peaks that represent cell type-specific genes and housekeeping genes, respectively. Moreover, the MG gene is usually more of a housekeeping gene than the SG gene, which is consistent with the results observed in previous work (Fig. 5F) [3]. Similar conclusions are reached with GM12878 Pore-C data (Supplementary Figure S16 available online at http://bib.oxfordjournals.org/).

**Figure 6:**
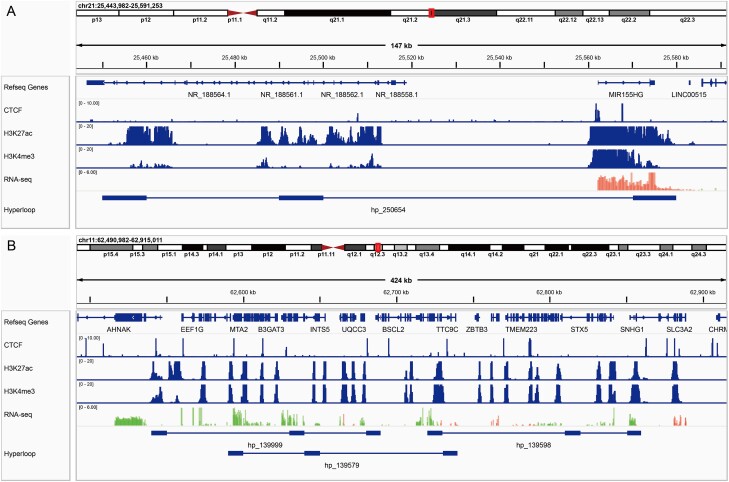
Examples of single gene hyperloop (A) and multiple gene hyperloop (B). Hyperloops are detected from GM12878 Pore-C data at 10 kb resolution. The H3K27ac and H3K4me3 tracks indicate the positions of enhancer and promoter respectively. The thick solid lines in the Hyperloop track represent loop anchors, and the thin solid lines connecting loop anchors represent a hyperloop. These snapshots were generated by Integrative Genomics Viewer (IGV) (https://igv.org).

Figure 6 shows examples of hyperloops involving multiple regulatory elements. Figure 6A shows a single gene hyperloop, in which H3K27ac is strongly enriched in the first two loop anchors, implying that each of the loop anchors contains a strong enhancer. The third loop anchor is enriched with a strong H3K4me3 signal and has a high RNA-seq signal, indicating that this anchor can correspond to an expressed gene and its promoter. Under the regulation of these two enhancers, the *MIR155HG* gene is highly expressed. This example illustrates that at the single-molecule level, multiple enhancers can indeed regulate the same promoter at the same time, which further indicates the important role of hyperloops in gene regulation and expression. Figure 6B shows three multiple-gene hyperloops, each of which contains highly expressed genes near the loop anchors. These genes may be clustered together in space to form a transcription factory, which coordinates gene expression. Together, HyperloopFinder can efficiently and effectively find these interactions from multi-way chromatin interaction data, thus providing candidates for the study of cooperative regulatory relationships between multiple genes and multiple regulatory elements.

## Discussion

In this work, we defined hyperloops as multi-way chromatin contacts that are statistically significant and connected by pairwise chromatin loops. Hyperloops are the generalization of chromatin loops that connect more than two genomic loci simultaneously. Furthermore, we developed the HyperloopFinder to detect hyperloops efficiently and effectively by utilizing an efficient frequent pattern mining algorithm and constructing a background model to predict the expected interaction frequency and estimate the statistical significance of hyperloop candidates. This model exclusively incorporates the impact of the random polymer looping effect and technical biases on the interaction sites of multi-way contacts. The results show that these two factors alone adequately accounted for a median of 83% of the variance in the multi-way contact frequency. This means that these two factors, especially the random polymer looping effect, are major determinants of the interaction frequency of multi-way contacts, and it is not enough to measure the importance of multi-way contacts only by considering the interaction frequency. This also suggests that most multi-way contacts are just random combinations of random pairwise contacts and have no actual biological meaning.

Then, we evaluated the hyperloops by DNA seqFISH+ data, and the results show that the fluorescence sites corresponding to the interaction sites of the hyperloop tend to be spatially colocalized compared to control samples. We then found that hyperloops are often located within open chromatin compartments and often within a few TADs, consistent with recent findings. Then, we explored the relationship between the hyperloop and gene regulation and found that the hyperloop can function as a scaffolding for enhancers and promoters to regulate gene expression, consistent with recent studies.

Similar methods have been proposed to detect significant multi-way contacts in Quinodoz’s work [13]. Quinodoz’s method enumerates all multi-way contacts at 1 MB resolution and computes the expected frequency of multi-way contacts by randomly sampled regions across the genome containing the same linear genomic distances as control samples. In contrast, our method uses frequent pattern mining algorithms, FP-growth, and a binomial model to predict the interaction frequency of multi-way contacts, which is more efficient and can be applied to a higher resolution. Furthermore, by incorporating the modeling of technical biases, HyperloopFinder significantly enhances overall accuracy compared to our baselines without such modeling. Additionally, it generates candidates based on pair-wise loops, thereby substantially reducing the number of candidates.

Hyperloop is relevant to other biological concepts, including super-enhancers, transcription factories, and chromatin hubs. Super-enhancers are clusters of multiple enhancers that regulate gene expression related to cell identity [36–38]. The constituent enhancers within a super-enhancer work in an additive, synergistic, or redundant manner. Detecting super-enhancers and target genes from multi-way contacts is a potential application of HyperloopFinder. Transcription factories are nucleus organizations where multiple genes are transcribed together [3, 39], which can correspond to the multigene hyperloops we detected. Chromatin hubs are clusters of chromatin fragments that interact with each other [40]. However, previous studies on chromatin hubs were limited to population-level data, such as bulk Hi-C or ChIA-PET. Therefore, the hyperloops we defined here refer specifically to the chromatin hubs detected from multi-way interaction data as a generalization of the concept of chromatin loops.

In summary, our work has the following contributions: First, we give the precise definition of the hyperloops and the hyperloop detection problem. Second, we built a binomial model to explain the formation process of random multi-way contacts. Third, we combined frequent pattern mining algorithm FP-Growth and hypothesis testing of binomial models to give an efficient and effective hyperloop detection method: HyperloopFinder. Fourth, we modeled the technical biases of multi-way contacts, and this method can be used to eliminate the technical biases of SPRITE or Pore-C data. In addition, we discussed how to perform multiple hypothesis test correction in hyperloop detection and then proposed the grouped Benjamini–Hochberg procedure (Supplementary Text). This method can be used for other loop detection methods. In summary, our work contributes novel insights into higher-order chromatin structures and functions and has the potential to enhance our understanding of transcriptional regulation and other cellular processes.

## Methods

### Data availability

SPRITE data of the mESC and GM12878 cell lines were downloaded from the NCBI database (accession: GSE114242). Pore-C data were downloaded from the NCBI database (accession: GSE149117). DNA seqFISH+ data are available at Zenodo (https://zenodo.org/record/3735329). Mouse gene expression data were downloaded from the Expression Atlas (https://www.ebi.ac.uk/gxa/experiments/E-MTAB-3578/Results). TADs and A/B compartments detected from Hi-C data of the GM12878 cell line were downloaded from the NCBI database (accession: GSE63525). All other relevant data are available upon request.

### Definition of Hyperloop detection problem

The chromosome $N$ is divided into $n$ bins equally. The length of each bin is the resolution of the data. Let $B=\left\{{b}_1,{b}_2,\dots, {b}_n\right\}$ be the set of all bins of this chromosome and $C=\left\{{c}_1,{c}_2,\dots, {c}_m\right\}$ be the set of all multi-way interaction clusters obtained from sequencing technologies such as SPRITE and Pore-C. Each cluster ${c}_i$ contains a subset of bins chosen from set $B$. A subset of bins is termed a hyperloop candidate. If a hyperloop contains $k$ bins, it is called a *k-hyperloop*. The frequency of the hyperloop is defined as the number of clusters that contain a particular hyperloop. Mathematically, the frequency of the hyperloop $hp$, $Freq(hp)$ can be defined as follows:


$$ \mathrm{Freq}(hp)=\left|\left\{{c}_i\left| hp\right.\subseteq{c}_i,{c}_i\in C\right\}\right|, $$

where the symbol $\mid \cdot \mid$ denotes the number of elements in a set. Figure 1A is an example of a multi-way interaction dataset with 10 bins and five clusters. Figure 1D shows an example of a hyperloop $\left\{{b}_0,{b}_2,{b}_4,{b}_8\right\}$ whose frequency is 4.

Hyperloop detection problem can be formally stated as follows: Given a set of multi-way chromatin interaction clusters $C$, find all multi-way contacts whose frequency $\ge minfreq$, whose interaction bins are connected by pairwise loops, and whose interaction frequencies are significantly higher than expected interaction frequencies, where $minfreq$ is the threshold of frequency.

We developed HyperloopFinder to detect hyperloops. Figure 1 overviews the main steps of HyperloopFinder, and the following subsections describe the details of these steps.

### Generating contact matrix from multi-way interaction clusters

Hi-C data can be presented as a contact matrix whose elements are the contact frequency between row and column bins. Hi-C data can be regarded as multi-way chromatin interaction data that each cluster just has two interaction sites. Multi-way clusters that length of $k$ can be decomposed to $$\left(\!\!\begin{array}{l}k\\{}2\end{array}\!\!\right)=k\left(k-1\right)/2$$ pairwise contacts. In this way, multi-way interaction data can be transformed into pairwise contacts, and we can process it using the tools developed for Hi-C data. In this step, we modified the script provided by the SPRITE data pipeline [13].

Similar to Hi-C data, the frequency of interactions between each pair of bins is influenced by factors such as GC content, fragment length, and mappability [29–31]. Therefore, we employed the ICE normalization algorithm, similar to the standard processing pipeline for Hi-C data, to correct these biases [29].

### Detecting pairwise chromatin loops

FitHiC2 [9, 10] is used to detect pairwise loops in our pipeline. The background frequencies of random polymer interactions are modeled using a binomial distribution, followed by hypothesis testing with observed interaction frequencies to assign statistical significance to chromatin contacts. Besides the loops detected by our pipeline, HyperloopFinder can accept the pairwise loops detected by other methods, such as HiCCUPS [4], or detected from another dataset, such as Hi-C, Micro-C [41], and ChIA-PET.

### Mining frequent multi-way contacts

Counting the frequency of pairwise contacts is easy and has a low computational cost because we just need to add each contact to corresponding matrix cells. However, counting the frequency of hyperloops is difficult. If the number of bins is $n$, we must construct a $n\times n$ matrix to restore the frequency for pairwise contacts. However, for the *k-hyperloop*, we need to construct a ${n}^k$ tensor to restore the frequency if we process it like Hi-C data. If the maximum size of the hyperloop is $k$, we need to construct $k-3$ tensors and restore ${n}^k+{n}^{k-1}+\dots +{n}^3={n}^{k+1}-{n}^2 -n-1$ numbers. Hi-C data needs much memory and computational resource overhead when the resolution is extremely high. So, processing the multi-way interaction data in a manner like Hi-C will be more difficult.

To improve the efficiency of counting the frequency of multi-way contacts, we modeled it as a frequent pattern mining problem and utilized an efficient algorithm, FP-growth [27, 28, 42, 43]. FP-growth utilizes the Apriori Property: if an item set is frequent, all its subsets must also be frequent. Specifically, if a *k-hyperloop* is frequent, all its subsets must also be frequent. On the other hand, if *k-hyperloop* is not frequent, then all its supersets must also be not frequent. So, we just need to count the frequency of the hyperloop, whose subsets are also frequent. In this bottom-up fashion, we reduced the search process of interactions whose subsets are not frequent. In our pipeline, we used the FP-growth program (version 6.21) which can be downloaded from https://borgelt.net/fpgrowth.html.

### Testing connectivity

Let $G\left(V,E\right)$ presents a hyperloop candidate where $V$ is the interaction bins of the hyperloop and $E$ is pairwise loops detected by FitHiC2 or other methods. If the Graph $G$ is connective, the hyperloop candidate is left to test significance at the next steps.

### Learning the distance distribution of pairwise contacts

Using a single distance distribution of pairwise contacts to estimate the expected interaction frequency for multi-way contacts of different sizes will result in poor performance (Supplementary Figure S17 available online at http://bib.oxfordjournals.org/). Therefore, we learned a corresponding distance distribution for each hyperloop size. Specifically, we measured the weight of each pairwise contact based on the number of times this contact is used to build multi-way contacts of a particular hyperloop size. Specifically, we decomposed each cluster $C=\left\{{c}_1,{c}_2,\dots, {c}_m\right\}$ to ${C}_n^k$ subsets of size $k$, $n$ is the number of bins of this cluster and $k$ is the size of subsets. Then, these subsets are further decomposed into ${C}_k^2$ pairwise contacts. Finally, we decomposed all clusters into pairwise contacts in this two-step manner and obtained the distance distribution of the pairwise contacts for estimating the expected interaction frequency of multi-way contacts.

### Background model and significance estimating for hyperloops

Assuming that multi-way contacts are formed by randomly linking pairwise contacts of different lengths, we proposed a model for explaining the formation of multi-way contacts:

We first put all the pairwise contacts into a pool. If we randomly sample a $loop\left(A,B\right)$, its length will follow a distribution $P\left( dist\left(A,B\right)\right)$, where $dist\left(A,B\right)$ is the length of this loop. To generate a *4-hyperloop*, we just need to sample a start point *A*, sample 3 pairwise loops in the pool, and join them together one by one.

We explain our model with an example of $hp(ABCD)$ (Fig. 2A). This hyperloop has four interaction sites $\left\{A,B,C,D\right\}$. First, we randomly sampled one start site $A$ with the probability $\frac{1}{n}$. Second, knowing node $A$, randomly select a second site $B$ with probability ${p}_1=P\left(B/A\right)=P\left( dist\left(A,B\right)\right)$ according to the distance distribution of the pairwise contacts. Third, knowing node $B$, randomly select a third site $C$ with probability ${p}_2=P\left(C/ AB\right)=P\left( dist\left(B,C\right)\right)$. Last, knowing the node $C$, randomly select a fourth site $D$ with probability ${p}_3=P\left(D/ ABC\right)=P\left( dist\left(C,D\right)\right)$. Together, we randomly sampled the hyperloop with the probability:


\begin{align*}P(ABCD)&=P(A)P\left(B/A\right)P\left(C/ AB\right)P\left(D/ AB C\right)\\ &=P(A)P\left(B/A\right)P\left(C/B\right)P\left(D/C\right)\\ &=P(A)P\left( dist\left(A,B\right)\right)P\left( dist\left(B,C\right)\right)P\left( dist\left(C,D\right)\right)\\ &=\left(1/n\right)\times{p}_1\times{p}_2\times{p}_3\end{align*}

So, the expected interaction frequency of $hp(ABCD)$ is


$$ \lambda =P(ABCD)\times N, $$

where $N$ is the total number of random experiments. We can estimate $N$ from multi-way interaction clusters $C$ by $$N=\sum \limits_{i=1}^m\left(\!\!\begin{array}{c}\left|{c}_i\right|\\{}k\end{array}\!\!\right)$$, where $k$ is the size of hyperloop and $k=4$ in our example, $\left|{c}_i\right|$ is the size of the cluster ${c}_i$, $$\left(\!\!\begin{array}{c}\left|{c}_i\right|\\{}k\end{array}\!\!\right)$$ is the number of combinations when we choose $k$ bins from $\left|{c}_i\right|$ bins.

We can get the probability that the observed frequency is *k* when this hyperloop can be sampled with the probability *p* in one experiment by a binomial distribution:


$$ \Pr \left(\!\! K=k\right)=\left(\begin{array}{l}N\\{}k\end{array}\!\!\right){p}^k{\left(1-p\right)}^{N-k}. $$

We can get the p-value as the probability that the observed frequency is equal to or greater than $k$:


$$ P\left(K\ge k\right)=\sum \limits_{i=k}^N\Pr \left(K=i\right). $$

Last, we applied multiple testing corrections using the Benjamini-Hochberg procedure to estimate q-values [44].

When we calculate the probability of generating a hyperloop, we only consider the formation of loops between adjacent bins. Let us use graph theory to illustrate the rationality of this approximation. All the bins comprising the hyperloop are considered as nodes, while the loops formed between the bins are regarded as edges. The hyperloop represents the spatial co-location of all bins, thus forming a complete graph where each pair of bins is connected by an edge. As spatial positions exhibit transitivity, the formation of the hyperloop merely requires the bins to form a connected graph, and then, following transitivity, indirectly connected edges together form a complete graph. Therefore, any graph where the nodes are connected can explain the generation process of this hyperloop. However, with the increase in the number of nodes, the potential configurations grow exponentially, leading to greater complexity. Considering the distance effect—where the interaction frequency decreases as the distance between loci increases, following a power-law relationship—we can focus on the most probable subgraph. This implies assigning a weight to each edge of the complete graph based on the log-transformed interaction probability according to the genomic distance. By seeking the maximum spanning tree, we can find the subgraph that maximizes the probability of connecting these bins, represented by the path that sequentially connects these bins. Our approximation simplifies the calculation of probability, ensuring the efficiency and scalability of the algorithm.

### Modeling the technical biases

Besides the formation of loops, TADs, compartments, or other higher-order structures, technical biases such as GC content and mappability will influence the interaction frequency of Hi-C or SPRITE data. To model technical biases, we used the ICE normalization procedure [29] to compute the bias of each bin, which is also used by Fit-Hi-C [9, 10].

From the pairwise contact matrix $M$, we can get a factorizable bias ${B}_i$ for each bin by solving the following equations:


$$ {M}_{ij}={B}_i{B}_j{M}_{ij}^{\ast }, $$

$$ \sum \limits_{i=1,\mid i-j\mid >1}^n{M}_{ij}^{\ast }=1, $$

where ${M}_{ij}$ is the observed frequency in data, ${B}_i$ and ${B}_j$ are the bias for bin $i$ and bin $j$, $n$ is the number of bins, ${M}_{ij}^{\ast }$ is the normalized frequency that has no technical bias. The second equation ensures equal visibility across all bins and eliminates the technical bias in a manner that does not consider the specific source of biases.

Let ${b}_i={B}_i/\overline{B}$, ${b}_j={B}_j/\overline{B}$, ${m}_{ij}={M}_{ij}^{\ast }{\overline{B}}^2$, where $\overline{B}=\frac{1}{n}\sum \limits_{i=1}^n{B}_i$, then we can transform the first equation to ${M}_{ij}={b}_i{b}_j{m}_{ij}$. In this way, the bias value is 1 when there is no bias for the bin and ${m}_{ij}={M}_{ij}$ when there is no bias for the bin $i$ and bin $j$.

When we calculated the expected interaction frequency $\lambda$ and sample probability $p$, we did not consider the bias for each bin in the last section. Now we can give the corrected $\lambda$ and $p$: ${\lambda}^{\ast }=\lambda \times{b}_1\times{b}_2\times \dots \times{b}_k$, ${p}^{\ast }=p\times{b}_1\times{b}_2\times \dots \times{b}_k$, where $k$ is the size of the hyperloop.

We used the coefficient of determination ${R}^2$ to evaluate the goodness of the prediction of expected interaction frequency:


$$ {R}^2=1-\frac{S{S}_{res}}{S{S}_{tot}}=1-\frac{\sum \limits_{i=1}^n{\left({y}_i-{\hat{y}}_i\right)}^2}{\sum \limits_{i=1}^n{\left({y}_i-\overline{y}\right)}^2}, $$

where ${y}_i$ is the observed value, ${\hat{y}}_i$ is the expected value, $\overline{y}$ is the mean of observed values, $S{S}_{res}$ is the sum of squared residuals, and $S{S}_{tol}$ is the total sum of squares.

**Key Points**

- Hyperloops are defined as multi-way chromatin contacts that are statistically significant and connected by pairwise chromatin loops.
- HyperloopFinder can detect hyperloops efficiently and effectively by utilizing an efficient frequent pattern mining algorithm and constructing a binomial model to predict the expected interaction frequency and estimate the statistical significance of hyperloop candidates.
- Our model revealed that most multi-way contacts can be formed by randomly linking the pairwise contacts adjacent to each other.
- Hyperloops are often located within open chromatin compartments and often within the same TAD.
- Hyperloops can function as scaffolding for enhancers and promoters to regulate gene expression.

## Supplementary Material

Supplementary_Figures_bbae341

Supplementary_Tables_bbae341

Supplementary_Text_bbae341

## Source code availability

A Python implementation of the HyperloopFinder has been deposited at GitHub https://github.com/GaoLabXDU/HyperloopFinder.
