# Supplementary material for: Statistical modeling and significance estimation of multi-way chromatin contacts with HyperloopFinder
Source: Brief Bioinform. 2024 Jul 14;25(4):bbae341. doi: 10.1093/bib/bbae341 (PMC11246602; doi:10.1093/bib/bbae341)
Supplement: Supplementary_Figures_bbae341 [file supplementary_figures_bbae341.docx]

Supplementary Figures


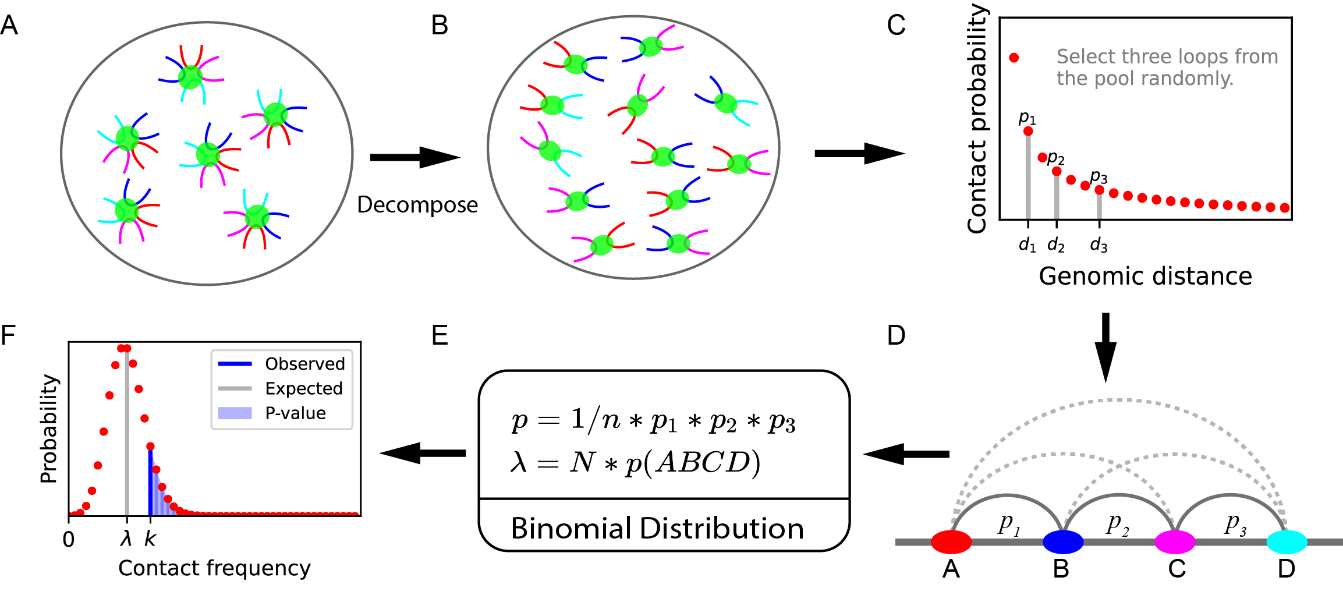


**Figure S1. Schematic illustration of the background model of HyperloopFinder.** (A) Molecular complexes linking multiple chromatin fragments. (B) Molecular complexes are decomposed to pairwise contacts. (C) Probability distribution of genomic distance if a pairwise loop is randomly selected. (D) Select three loops from the pool and sample a start point A randomly and connect them end to end, we can generate a 4-way contact. (E) Computing the expected interaction frequencies of hyperloops and building the binomial Models. (F) Significance estimating for hyperloops.


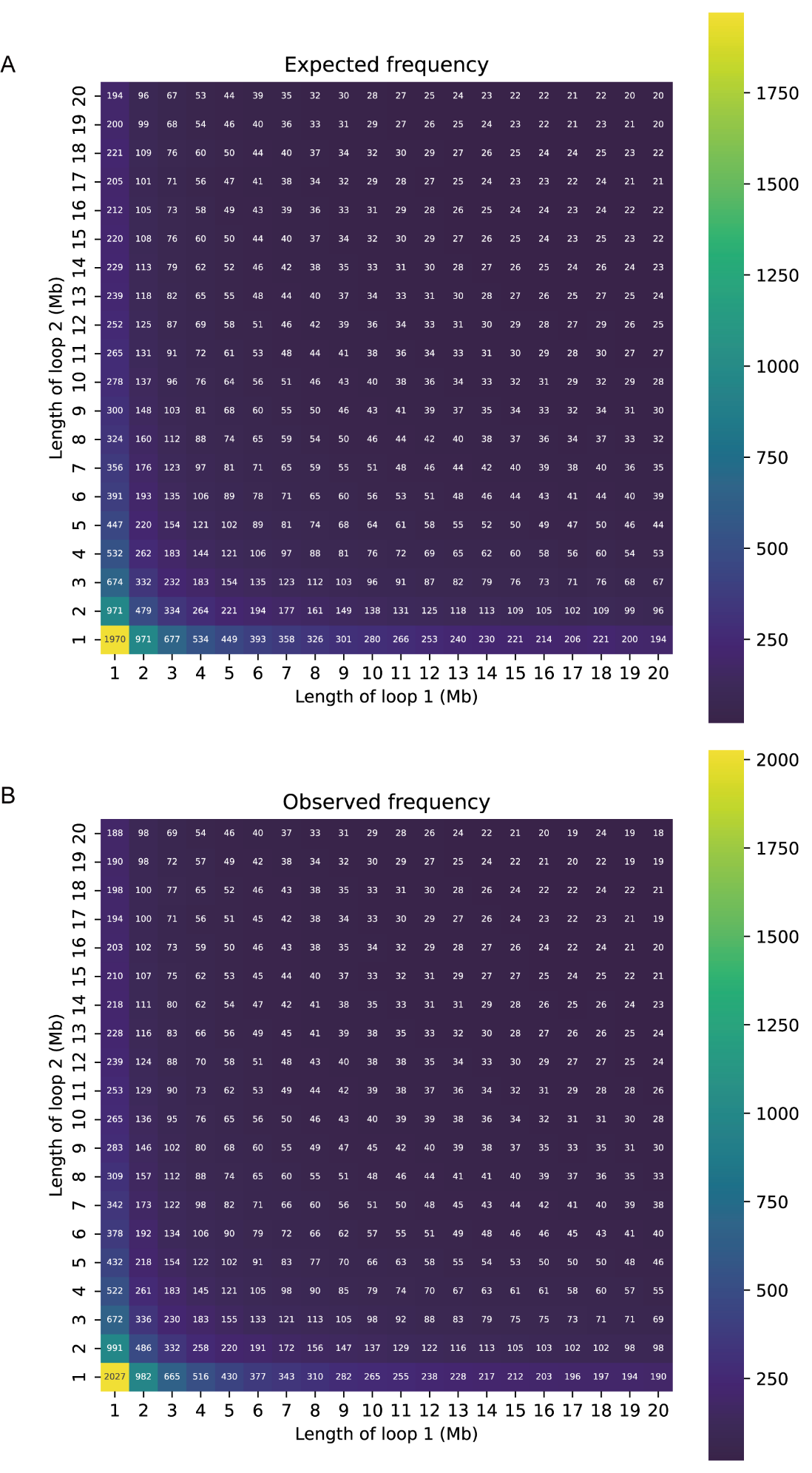


**Figure S2. Expected and observed interaction frequency of Chr1, GM12878 cell line, Pore-C data.** (A) Expected interaction frequency for different length pairs of loops. (B) Observed interaction frequency for different length pairs of loops.


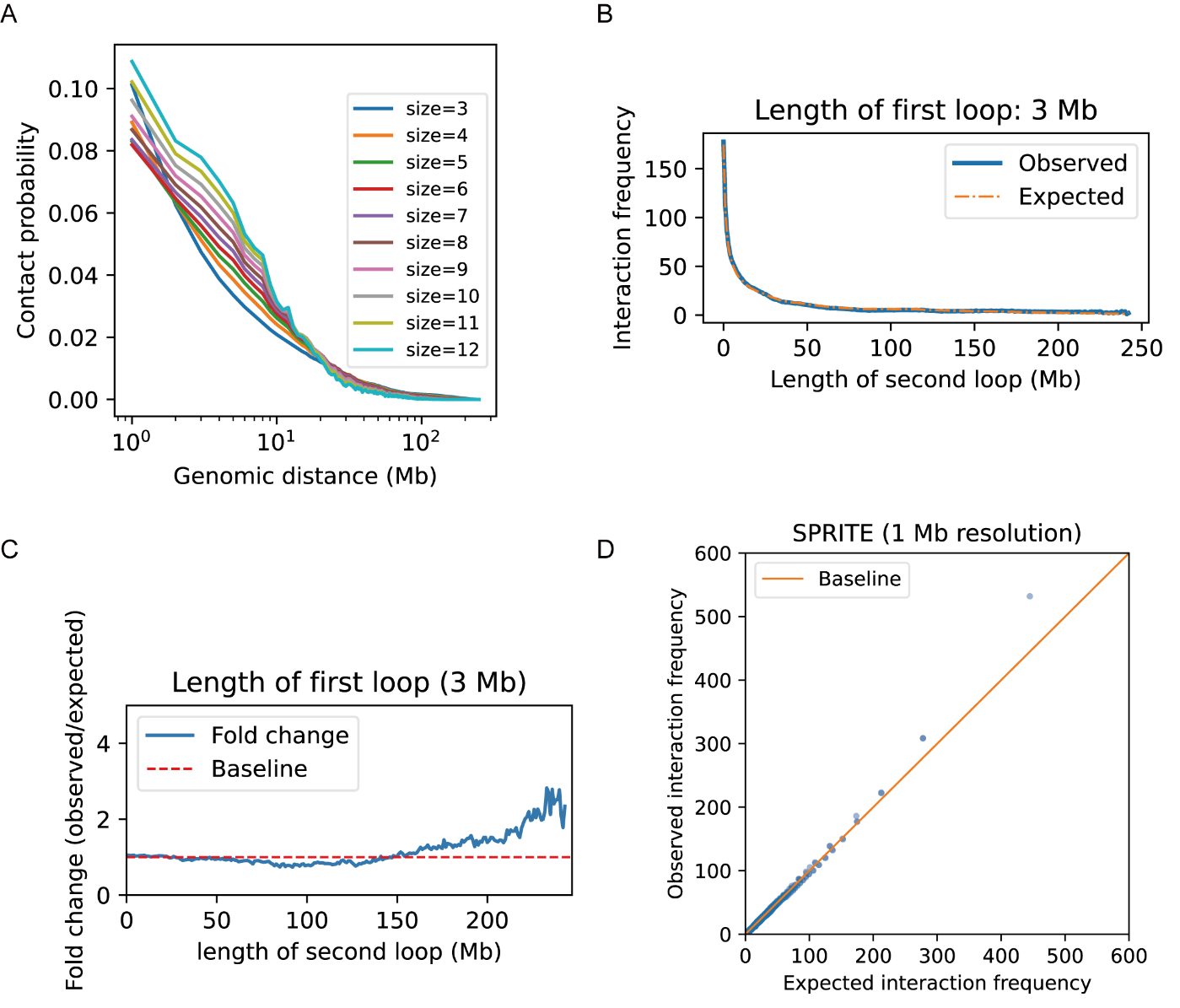


**Figure S3. Evaluation of background model with SPRITE data, GM12878 cell line.** (A) Genomic distance distribution of pairwise contacts for different sizes of multi-way contacts. (B) Fixed the length of the first loop to 3 Mb, observed and expected interaction frequency all decreased with the increase of the length of the second loop. (C) Fixed the length of the first loop to 3 Mb, fold enrichment between observed and expected interaction frequency with the increase of the length of the second loop. (D) Expected interaction frequencies are highly correlated with observed interaction frequencies.


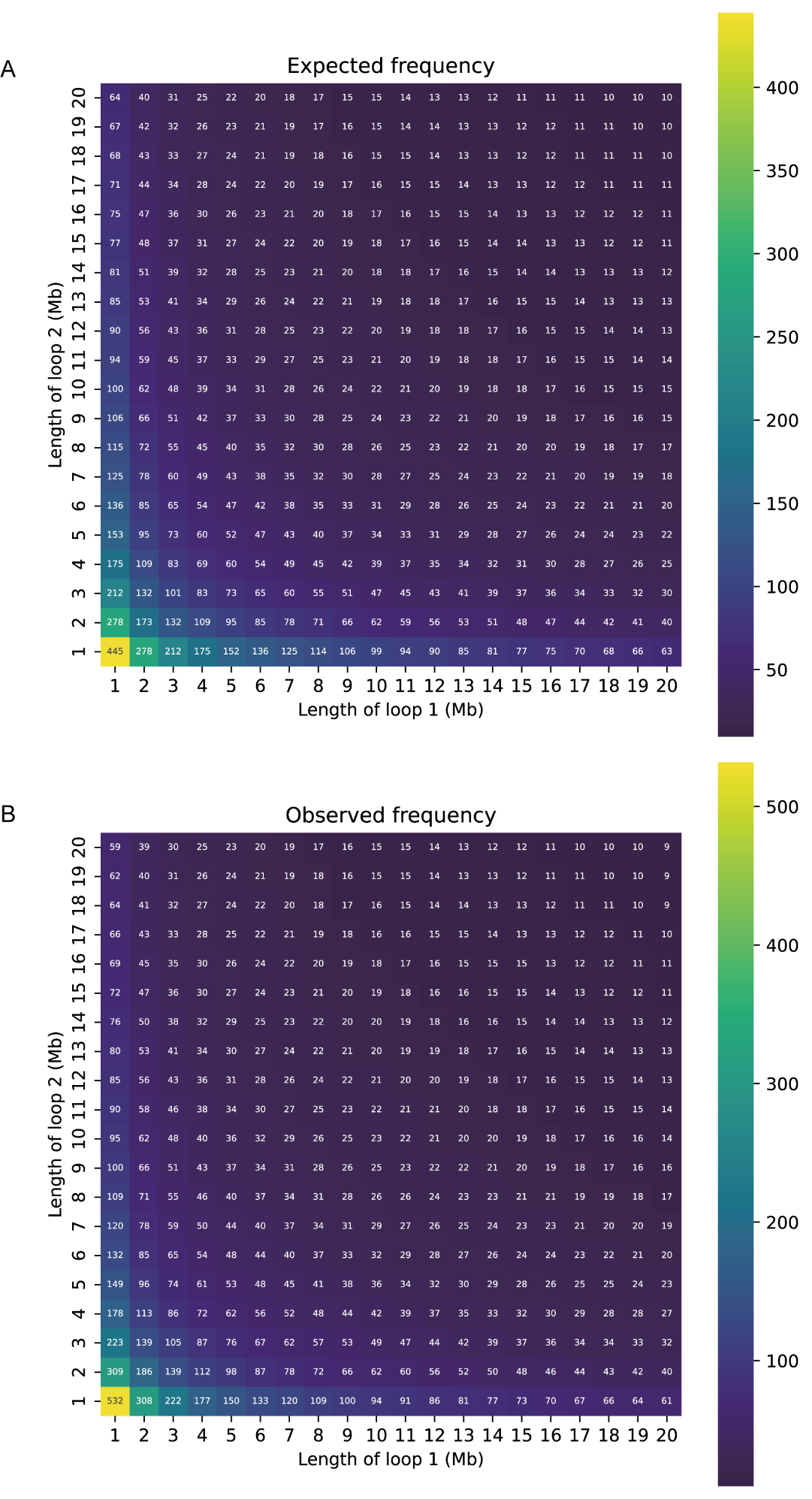


**Figure S4. Expected and observed interaction frequency of Chr1, GM12878 cell line, SPRITE data.** (A) Expected interaction frequency for different length pairs of loops. (B) Observed interaction frequency for different length pairs of loops.


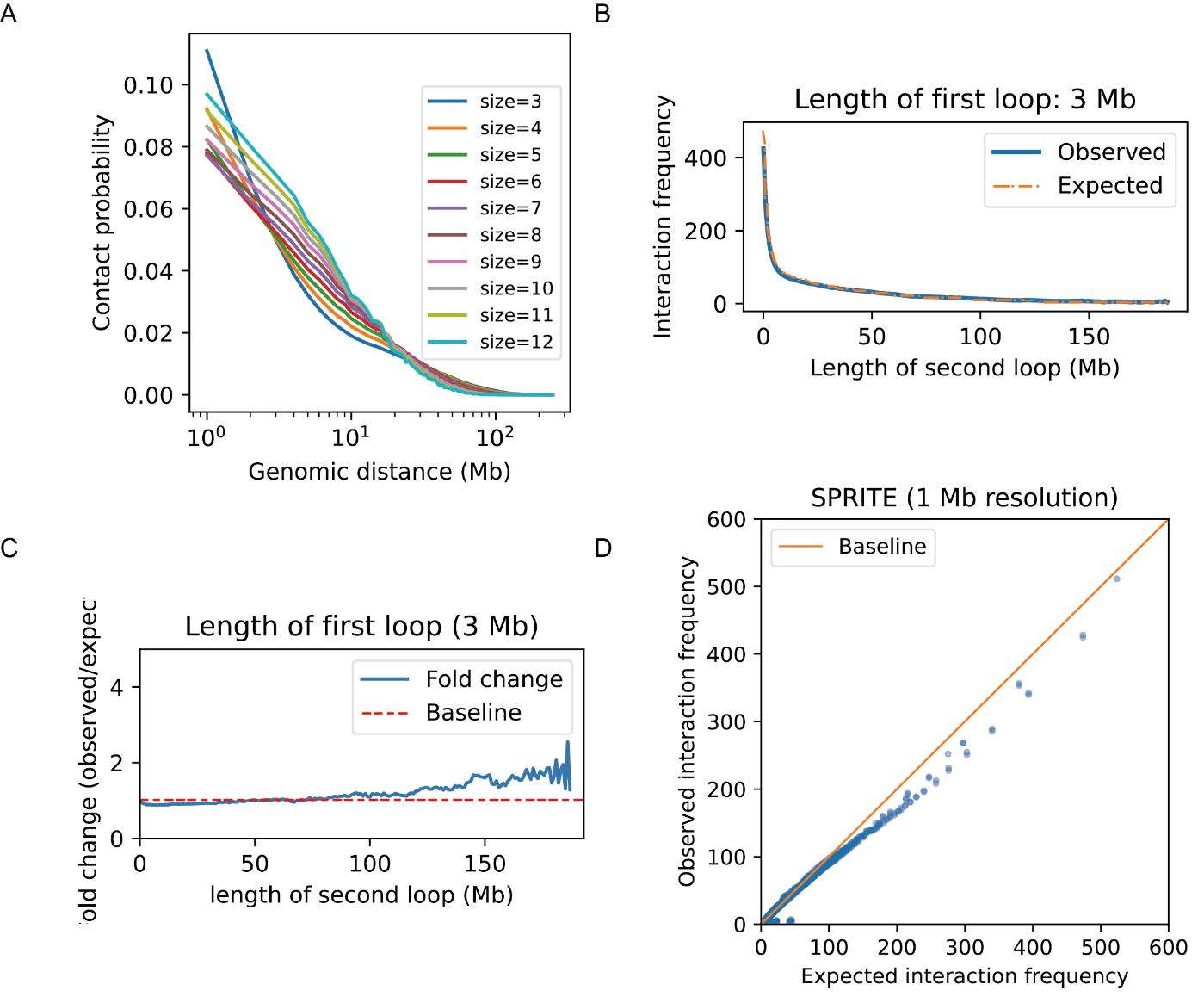


**Figure S5. Evaluation of background model with SPRITE data, mESC cell line.** (A) Genomic distance distribution of pairwise contacts for different sizes of multi-way contacts. (B) Fixed the length of the first loop to 3 Mb, observed and expected interaction frequency all decreased with the increase of the length of the second loop. (C) Fixed the length of the first loop to 3 Mb, fold enrichment between observed and expected interaction frequency with the increase of the length of the second loop. (D) Expected interaction frequencies are highly correlated with observed interaction frequencies.


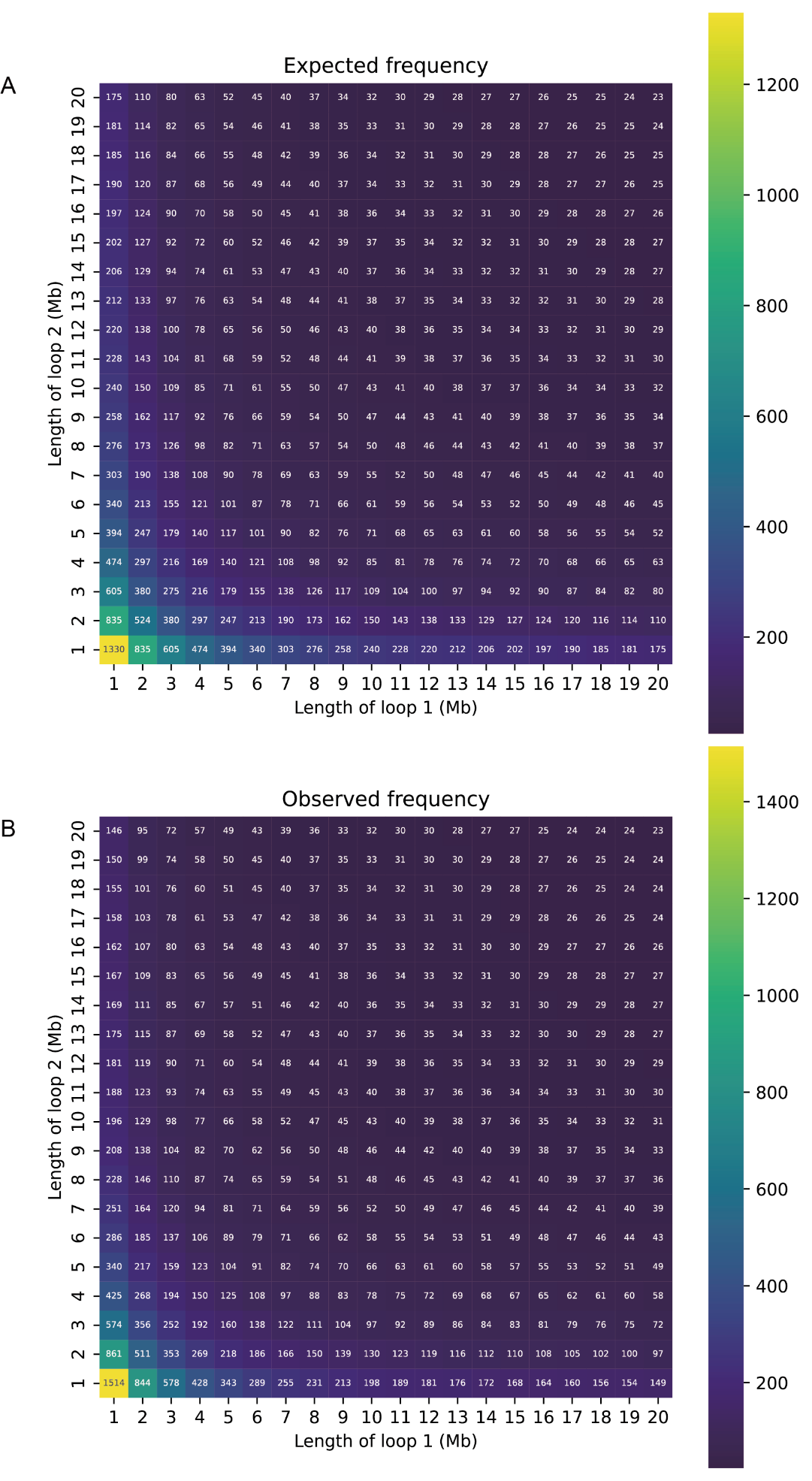


**Figure S6. Expected and observed interaction frequency of Chr1, mESC cell line, SPRITE data.** (A) Expected interaction frequency for different length pairs of loops. (B) Observed interaction frequency for different length pairs of loops.


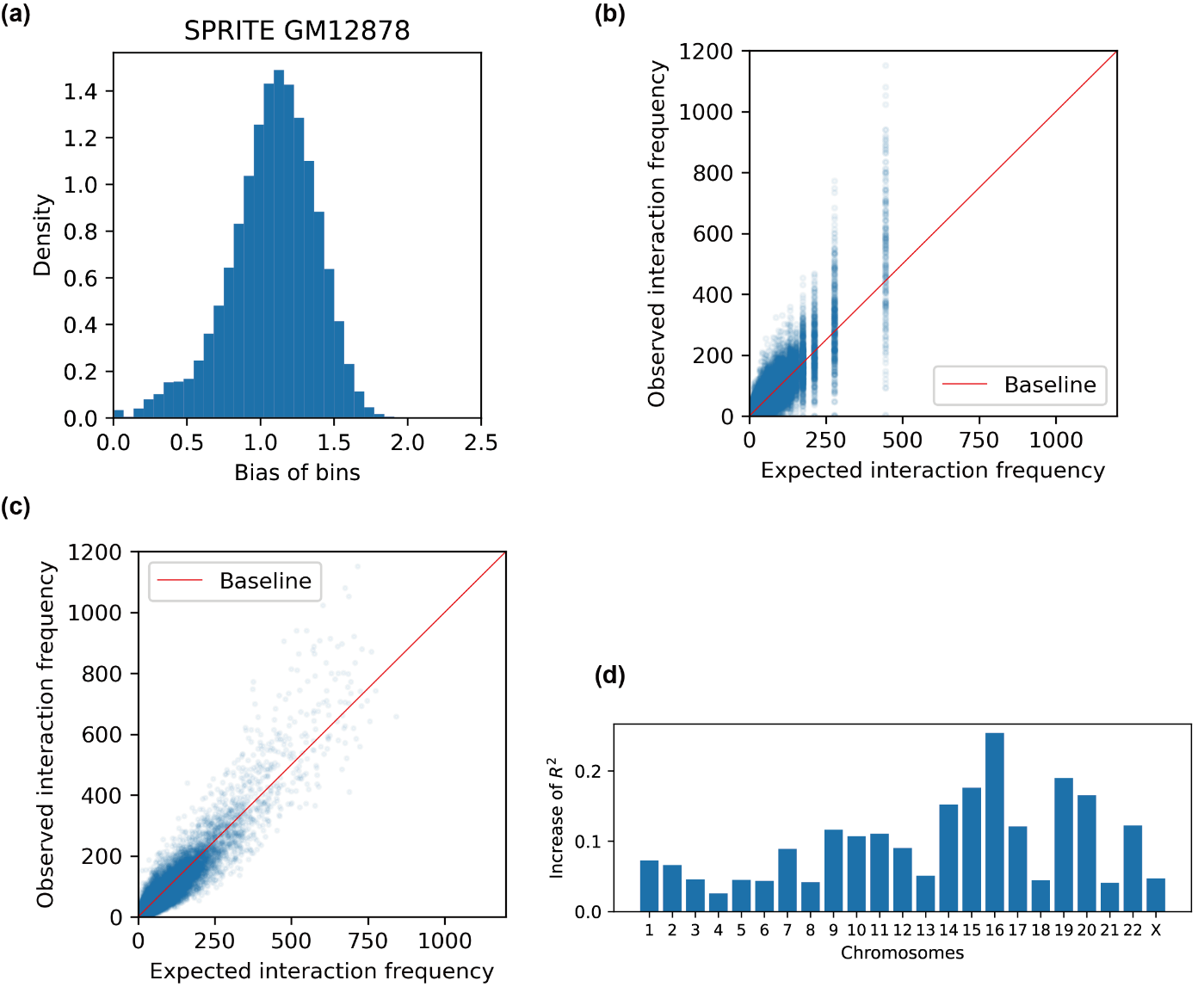


**Figure S7. Modeling the biases of SPRITE data, GM12878 cell line.** (A) Histogram showing the distribution of biases of bins, GM12878 cell line, SPRITE data, chr1, 1 Mb resolution. (B) Expected interaction frequency vs observed interaction frequency without bias modeling. (C) Expected interaction frequency vs observed interaction frequency with bias modeling. (D) Bar plot showing the increase in R^2^ after modeling the bias.


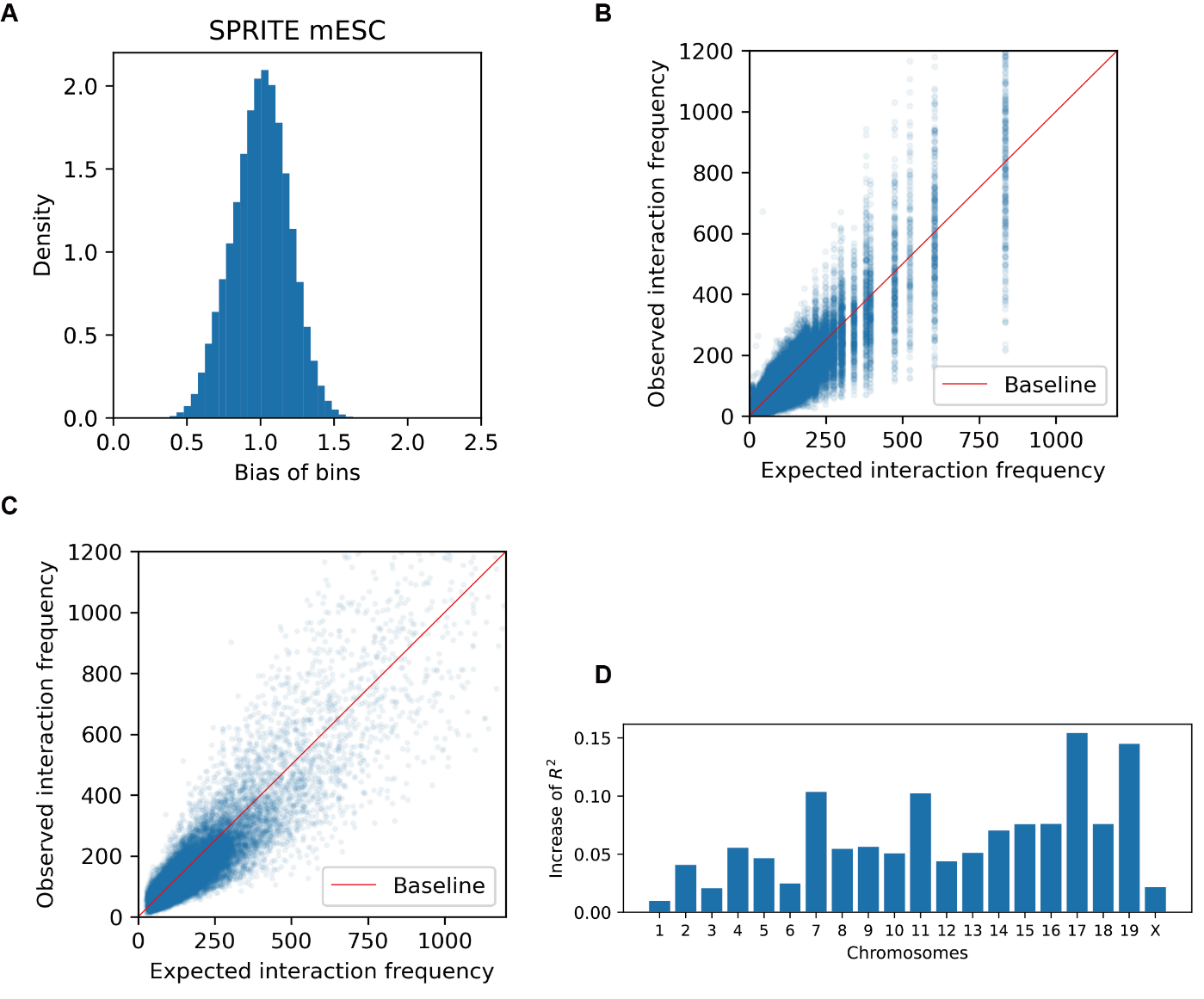


**Figure S8. Modeling the biases of SPRITE data, mESC cell line.** (A) Histogram showing the distribution of biases of bins, mESC cell line, SPRITE data, chr1, 1 Mb resolution. (B) Expected interaction frequency vs observed interaction frequency without bias modeling. (C) Expected interaction frequency vs observed interaction frequency with bias modeling. (D) Bar plot showing the increase in R^2^ after modeling the bias.


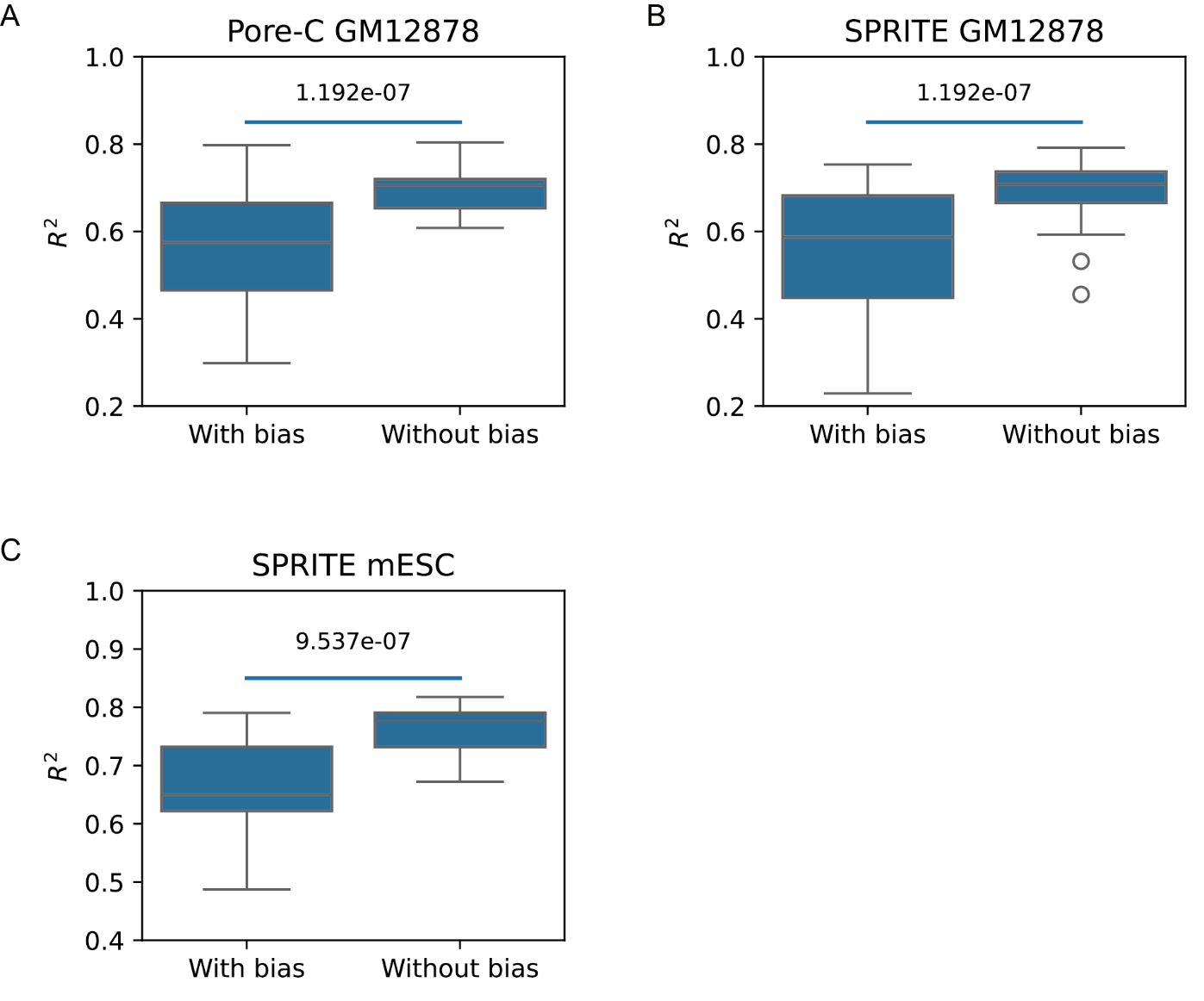


**Figure S9. R^2^ score compared between before/after bias modeling.** Before R^2^ scores are computed, expected and observed interaction frequency of hyperloops are log-transformed.

**Figure S10. Running time of HyperloopFinder for different steps and minimum support count setting.** GM12878 cell line, Pore-C data, chr1, and 25 kb resolution. Step 1: generating Hi-C heatmap, computing the biases of bins, and detecting pairwise loops. Step 2: binning and splitting the multi-way contacts to different chromosome files. Step 3: mining frequency pattern using FP-growth algorithm. Step 4: testing the connectivity of hyperloop candidates using pairwise loops detecting from step 1. Step 5: testing the significance of hyperloop candidates.

**Figure S11. False discovery rate.**


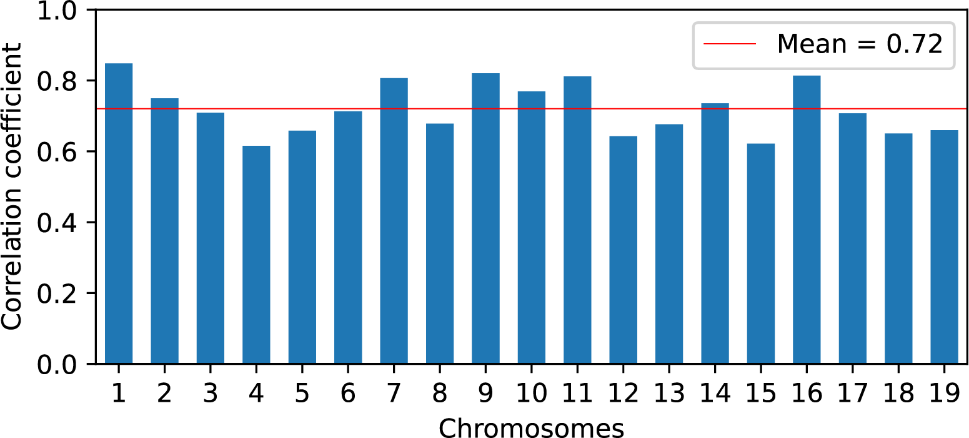


**Figure S12. Correlation coefficient between the interaction frequency of SPRITE data and the colocalization ratio of DNA seqFish+ data at different chromosomes.** The colocalization threshold is set to 250 nm.


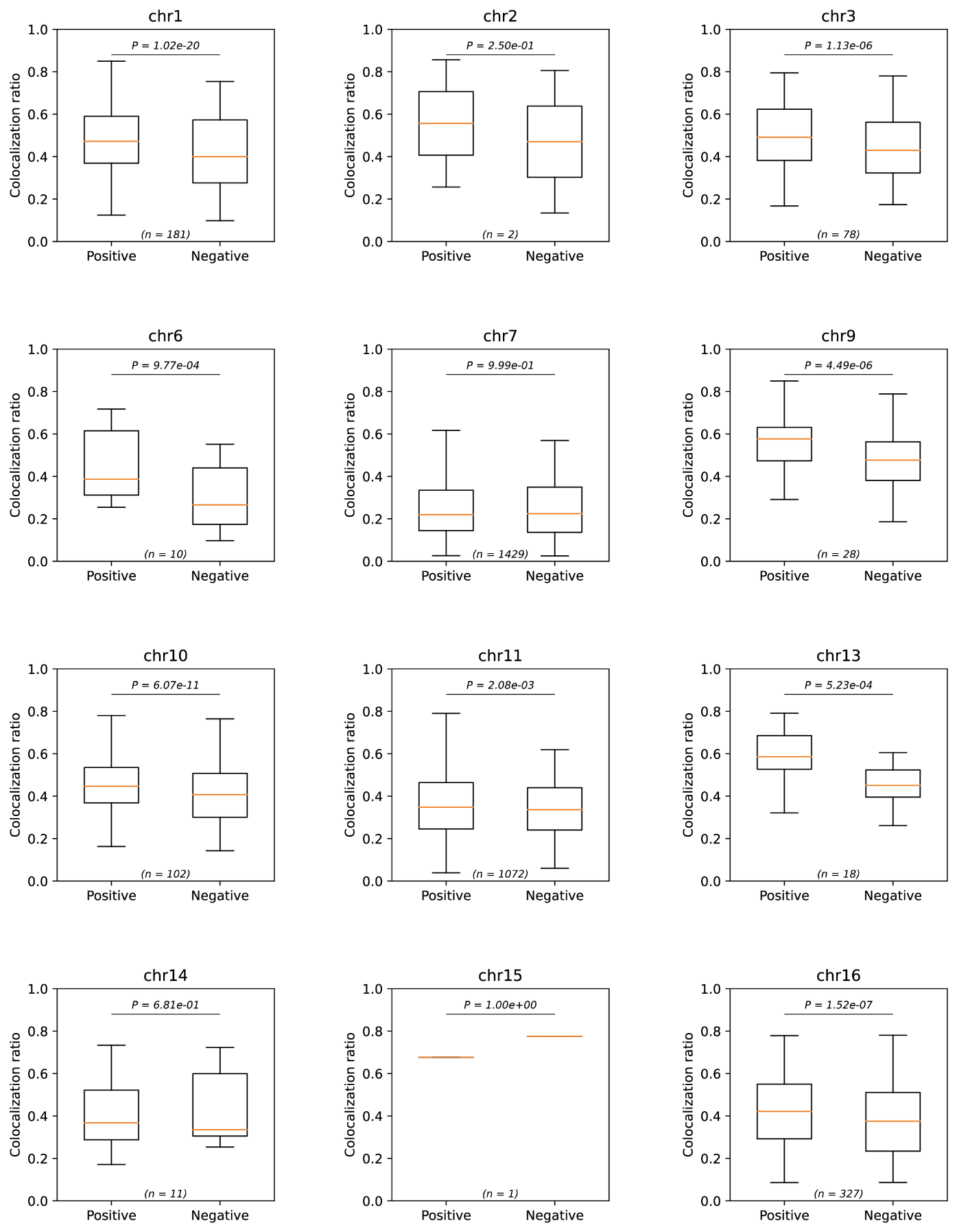


**Figure S13. Colocalization ratio compared between hyperloops and random shuffled samples.**

**
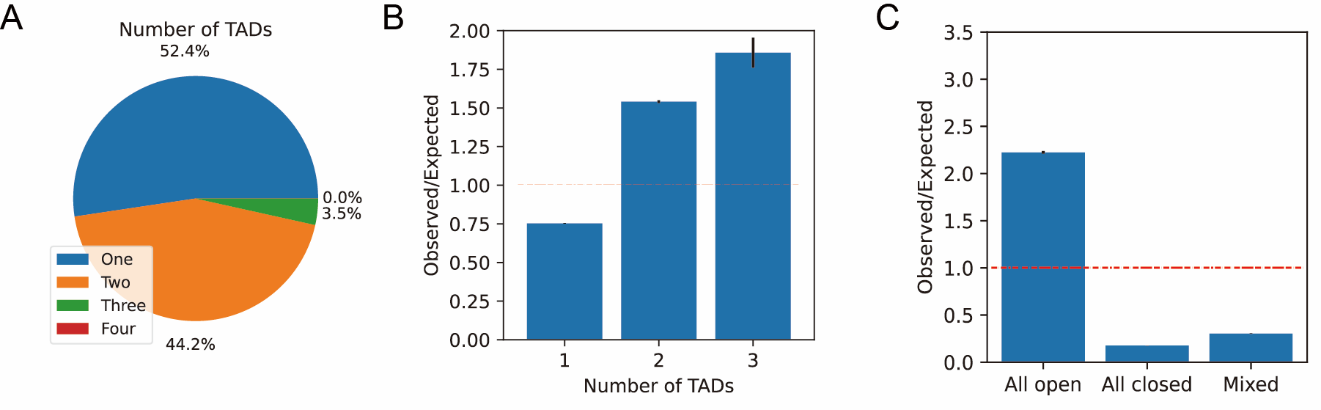
**

**Figure 14. Hyperloops and chromatin structures.** SPRITE data, mESC cell line. (A) Pie chart shows the percentage of hyperloops that span different TAD numbers. (B) Fold enrichment of the number of hyperloops that span different TAD numbers compared with random shuffled TADs. (C) Fold enrichment of the number of hyperloops that span different compartment patterns compared with randomly shuffled compartment labels.

**Figure S15. Number of genes involved in MG, SG, and BP hyperloops.** mESC cell line, SPRITE data. MG represents the multiple gene hyperloops, BP represents the basal promoter hyperloops, and SG represents the single gene hyperloops.


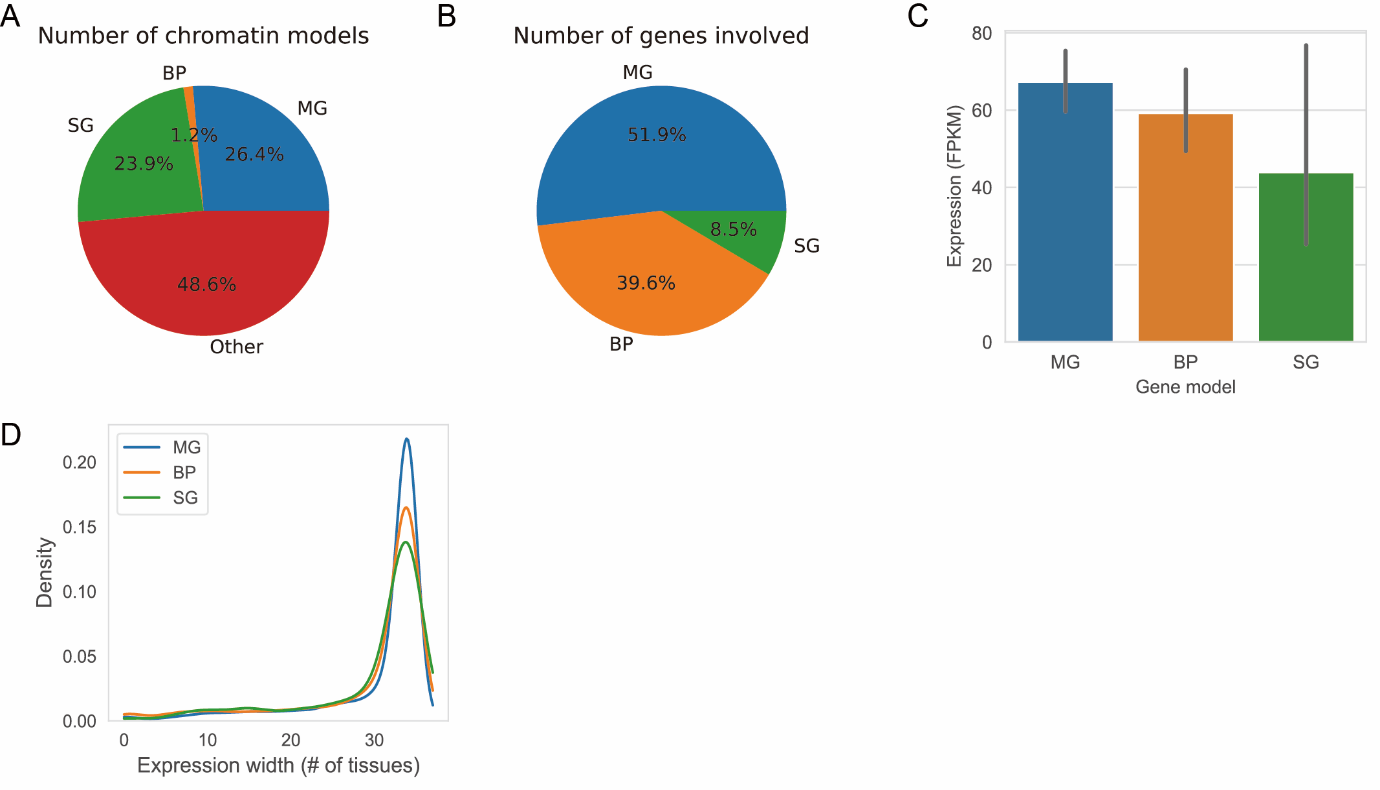


**Figure S16. Hyperloops and transcriptional regulation.** Pore-C data, GM12878 cell line. (A) Pie chart shows the percentage of hyperloops that belong to different chromatin models. MG represents the multiple gene hyperloops, BP represents the basal promoter hyperloops, SG represents the single gene hyperloops, and Other represents hyperloops that are not associated with gene expression. (B) Number of genes involved in MG, SG, and BP hyperloops. (C) Bar plots show the expression difference of genes associated with different chromatin models. (D) Expression width analysis of the genes that belong to different chromatin models.


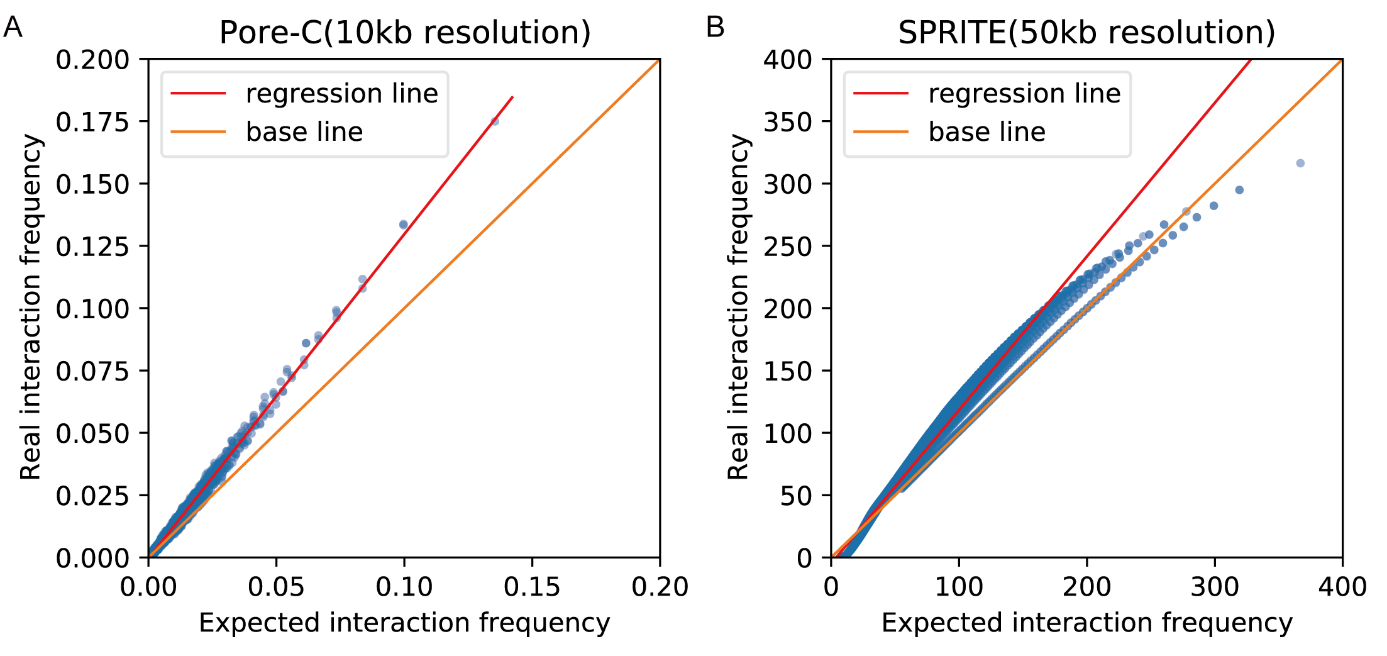


**Figure S17. Real vs Expected interaction frequency of Pore-C data (A) and SPRITE data (B).** The expected frequency is estimated by the background model of HyperloopFinder, but the universal distribution of pairwise loop length is used.

**Fig. S18. The number of significant loops tends to grow with the number of groups.**
