# Supplementary material for: Statistical modeling and significance estimation of multi-way chromatin contacts with HyperloopFinder
Source: Brief Bioinform. 2024 Jul 14;25(4):bbae341. doi: 10.1093/bib/bbae341 (PMC11246602; doi:10.1093/bib/bbae341)
Supplement: Supplementary_Tables_bbae341 [file supplementary_tables_bbae341.docx]

Supplementary Tables

**Table 1. The number of hyperloops at different resolutions, GM12878, Pore-C data**

| **size** | **10k** | **25k** | **50k** | **100k** |
| --- | --- | --- | --- | --- |
| **3** | 11492 | 318933 | 571142 | 421024 |
| **4** | 359 | 31370 | 275476 | 411860 |
| **5** | 0 | 0 | 17053 | 136010 |
| **6** | 0 | 0 | 0 | 5742 |
| **total** | 11851 | 350303 | 863671 | 974636 |

Parameters: max_cluster_size = 100, min_support_count = 3 for 10k, min_support_count = 5 for 25k, 50k, and 100k, loop_fdr_cut = 0.05, loop_upperbound = 2000000, loop_lowerbound = 0.

**Table 2. The number of hyperloops at different resolutions, GM12878, SPRITE data**

| **size** | **10k** | **25k** | **50k** | **100k** |
| --- | --- | --- | --- | --- |
| **3** | 387 | 23229 | 201551 | 190943 |
| **4** | 0 | 292 | 88185 | 344486 |
| **5** | 0 | 2 | 6157 | 169863 |
| **6** | 0 | 0 | 53 | 23111 |
| **7** | 0 | 0 | 6 | 1262 |
| **8** | 0 | 0 | 0 | 114 |
| **9** | 0 | 0 | 0 | 3 |
| **total** | 387 | 23523 | 295952 | 729782 |

Parameters: max_cluster_size = 50, min_support_count = 5 for 10k, min_support_count = 10 for 25k, 50k, and 100k, loop_fdr_cut = 0.05, loop_upperbound = 2000000, loop_lowerbound = 0.

**Table 3. The number of hyperloops at different resolutions, mESC, SPRITE data**

| **size** | **10k** | **25k** | **50k** | **100k** |
| --- | --- | --- | --- | --- |
| **3** | 21678 | 487649 | 751005 | 404772 |
| **4** | 2890 | 159021 | 1857024 | 1544955 |
| **5** | 0 | 3225 | 1191377 | 3534126 |
| **6** | 0 | 1 | 217455 | 3112064 |
| **7** | 0 | 0 | 16253 | 1103184 |
| **8** | 0 | 0 | 176 | 203196 |
| **9** | 0 | 0 | 0 | 20626 |
| **10** | 0 | 0 | 0 | 953 |
| **11** | 0 | 0 | 0 | 11 |
| **total** | 24568 | 649896 | 4033290 | 9923887 |

Parameters: max_cluster_size = 50, min_support_count = 5 for 10k, min_support_count = 10 for 25k, 50k, and 100k, loop_fdr_cut = 0.05, loop_upperbound = 2000000, loop_lowerbound = 0.

**Table 4.** **Overlap coefficient of hyperloops between different resolutions**

| **Resolution 1** | **Resolution 2** | **Pore-C GM12878** | **SPRITE GM12878** | **SPRITE mESC** |
| --- | --- | --- | --- | --- |
| **10k** | **25k** | 0.84 | 0.82 | 0.97 |
| **10k** | **50k** | 0.95 | 0.98 | 1.00 |
| **10k** | **100k** | 0.97 | 0.94 | 1.00 |
| **25k** | **50k** | 0.97 | 0.97 | 0.98 |
| **25k** | **100k** | 0.99 | 0.98 | 1.00 |
| **50k** | **100k** | 0.97 | 0.96 | 0.98 |
