# Supplementary material for: Statistical modeling and significance estimation of multi-way chromatin contacts with HyperloopFinder
Source: Brief Bioinform. 2024 Jul 14;25(4):bbae341. doi: 10.1093/bib/bbae341 (PMC11246602; doi:10.1093/bib/bbae341)
Supplement: Supplementary_Text_bbae341 [file supplementary_text_bbae341.docx]

Supplementary Text

Grouped multiple hypothesis testing

Multiple hypothesis testing for hyperloops is a necessary but challenging task. The search space of hyperloops grows exponentially as resolution increases, which leads to the need for an extensive amount of hypothesis tests simultaneously. Even strictly controlled errors of the first type of hypothesis testing (null hypothesis is rejected even though it is true) can produce many false positive results. Given the order of magnitude difference between positive results and the search space, measuring the significance of results using only p-values can result in a high false discovery rate ($FDR=\frac{false\_positives}{positives}$). Therefore, the false discovery rate must be controlled by multiple testing correction.

Benjamini–Hochberg procedure [1] is widely used to control FDR by calculating q-values:

$$q_{i}=\frac{p_{i}\times n}{\mathrm{rank}\left(p_{i}\right)},i=1,2,\ldots,n$$

where $n$ is the number of hypotheses, $p_{i}$ is the p-value of hypothesis $i$, $q_{i}$ is the q-value of hypothesis $i$, $rank(p_{i})$ is the order of $p_{i}$ among all p-values that the p-values are sorted in ascending order. The p-value represents the probability that the null hypothesis is true but rejects the null hypothesis, so $p_{i}\times n$ can be considered the number of false-positive samples. $rank(p_{i})$ can be considered as the number of samples rejected by the correction procedure after using $p_{i}$ as a cut-off point, that is, the number of positive samples. Therefore, after defining samples whose $q_{i}$ less than a specific q-value threshold as significant, the FDR can be controlled below this threshold.

However, controlling the false discovery rate using the usual Benjamini–Hochberg procedure in this task is difficult. Specifically, when the interaction distance is extremely far or many sites are taking part in the interaction, the expected interaction frequency of the hyperloop may be much less than 1, resulting in a high significance even if the observation frequency is only 1. Simultaneous correction of samples with large differences in expected interaction frequencies may produce many multi-way contacts with expectations much less than 1 but extremely low observation frequencies. At the same time, many multi-way contacts with extremely high observed interaction frequency but relatively low interaction distance are incorrectly rejected.

So, we divided candidate hyperloops into groups with similar numbers according to their maximum interaction distance (the distance between the first and the last bin) and then performed the Benjamini–Hochberg procedure in each group. Meanwhile, another benefit of this approach is that the significance of a hyperloop no longer depends on our limitations on the maximum distance of interactions.

Another detail worth noting is that we used the theoretical number of multi-way contacts instead of the number of candidates in the group for multiple hypothesis test corrections. This is because some interactions have frequencies of 0 or are either below the minimum support threshold we set or are filtered out by us in connectivity testing for pairwise contacts. If these potentially non-significant samples are not considered, it is difficult to penalize p-values enough to produce too many false positives. We used the following formula to calculate the theoretical number of *k-hyperloops* under the limitation of maximum interaction distance $d$: $N(d,k)=(n-d+1)\left(\begin{matrix} d-1 \\ k-1 \end{matrix}\right)+\left(\begin{matrix} d-1 \\ k \end{matrix}\right)$, where $n$ is the number of bins of a specific chromosome. Then, the theoretical number of *k-hyperloops* of a specific group is $N\left(d_{\max},k\right)-N\left(d_{\min},k\right)$, where $d_{max}$ and $d_{min}$ is the maximum and minimum distance limit of this group, respectively.

The results show that when the number of groups is set to about 20, the number of results tends to be stable, so we set the number of groups to 50 by default (Supplementary Fig. S18). For the same reason, we performed the same grouped correction for the pairwise loop detected by FitHiC2. Recently, we found that HiCCUPS uses a similar strategy called $\lambda$­­-$chunking$ in loop detection [2].

Processing of DNA seqFISH+ data

DNA seqFISH+ enables the imaging of thousands of targeted DNA loci in single cells and achieves a resolution of up to 25 kb in a local region. DNA seqFish+ screens DNA sequence fragments at equal intervals, labels different fragments with different color sequence tags, and then determines the spatial three-dimensional coordinates of each specific DNA sequence by fluorescence imaging. Thus, DNA seqFish+ data can provide three-dimensional spatial location coordinates and linear genomic coordinates of DNA sequences. However, due to technical limitations, the spatial coordinates of some DNA sequences are not captured by fluorescence imaging, and the missing rate reaches 50%. Because the genome of most organisms is polyploid, the same DNA sequence may have multiple coordinates in space, it is necessary to calculate the homologous chromosomes and fill in the missing DNA sequence spatial coordinates.

We used a greedy strategy to divide each fluorescence site into different chromosomes and linear interpolation to fill in the three-dimensional coordinates of missing fluorescent sites on chromosomes. The main steps are as follows:

Step 1: two fluorescent sites that are spatially distant (>1500 nm) and belong to one DNA sequence, and have the minimum region ID, that is, linear sequential encoding of genomic regions, are used as the initial and last nodes of the two homologous chromosomes.

Step 2: each DNA sequence is traversed in the order of region ID, and all fluorescent sites corresponding to this sequence are assigned to the two chromosomes by the distance from the last nodes. If there are more than two fluorescent sites at this location, only two are remained. The last nodes of both chromosomes are then updated. If only one fluorescence site responds to this region, only its corresponding last node is updated.

Step 3: We used the strategy of linear interpolation to fill in the three-dimensional coordinates of missing fluorescent sites on chromosomes.

Finally, about 50% of the missing loci were completed using linear interpolation, resulting in the spatial position coordinates of the consecutive DNA loci for each chromosome within 1.5 Mb size at 25 kb resolution.

Reference

1. Benjamini Y, Hochberg Y. Controlling the False Discovery Rate: A Practical and Powerful Approach to Multiple Testing. Journal of the Royal Statistical Society: Series B (Methodological) 1995; 57:289–300

2. Rao SSP, Huntley MH, Durand NC, et al. A 3D Map of the Human Genome at Kilobase Resolution Reveals Principles of Chromatin Looping. Cell 2014; 159:1665–1680
